# Genetic diversity, genetic structure and diet of ancient and contemporary red deer (*Cervus elaphus* L.) from north-eastern France

**DOI:** 10.1371/journal.pone.0189278

**Published:** 2018-01-05

**Authors:** Annik Schnitzler, José Granado, Olivier Putelat, Rose-Marie Arbogast, Dorothée Drucker, Anna Eberhard, Anja Schmutz, Yuri Klaefiger, Gérard Lang, Walter Salzburger, Joerg Schibler, Angela Schlumbaum, Hervé Bocherens

**Affiliations:** 1 LIEC UMR 7360, University of Lorraine - UFR Sci FA, Campus Bridoux, Metz, France; 2 Integrative Prehistoric and Archaeological Science (IPAS), University of Basel, Basel, Switzerland; 3 Archéologie Alsace, Sélestat & UMR 7041 ArScan - Archéologies environnementales - Maison de l’Archéologie et de l’Ethnologie, Nanterre, France; 4 CNRS UMR 7044 – ARCHIMEDE - Misha, Strasbourg, France; 5 Senckenberg Center for Human Evolution and Palaeoenvironment (HEP), University of Tübingen, Tübingen, Germany; 6 Zoological Institute, University of Basel, Basel, Switzerland; 7 26 A rue principale, Gries, France; 8 Dept of Geosciences (Biogeology), University of Tübingen, Tübingen, Germany; National Cheng Kung University, TAIWAN

## Abstract

In north-eastern France, red deer (*Cervus elaphus* L.) populations were rebuilt from a few hundred individuals, which have subsisted in remote valleys of the Vosges mountains, and to a lesser extent from individuals escaped from private enclosures; at present times, this species occupies large areas, mainly in the Vosges Mountains. In this study, we examined the population dynamics of red deer in the Vosges Mountains using ancient and contemporary mitochondrial DNA (mtDNA) from 140 samples (23 ancient + 117 modern) spanning the last 7’000 years. In addition, we reconstructed the feeding habits and the habitat of red deer since the beginning of agriculture applying isotopic analyses in order to establish a basis for current environmental management strategies. We show that past and present red deer in the Vosges Mountains belong to mtDNA haplogroup A, suggesting that they originated from the Iberian refugium after the last glacial maximum (LGM). Palaeogenetic analysis of ancient bone material revealed the presence of two distinct haplotypes with different temporal distributions. Individuals belonging to the two haplotype groups apparently occupied two different habitats over at least 7’000 years. AM6 correlates with an ecological type that feeds in densely forested mountain landscapes, while AM235 correlates with feeding in lowland landscapes, composed of a mixture of meadows and riverine, herb-rich woodlands. Our results suggest that red deer of north-eastern France was able to adapt, over the long term, to these different habitat types, possibly due to efficient ethological barriers. Modern haplotype patterns support the historical record that red deer has been exposed to strong anthropogenic influences as a major game species.

## Introduction

Red deer (*Cervus elaphus* L.) is currently one of the most widespread European ungulate species and its evolutionary history is relatively well established [1, 2, 3, 4, 5]. Molecular studies revealed the existence of at least three distinct mitochondrial DNA (mtDNA) lineages (so called matrilines) in Europe reflecting the main refugia during the Last Glacial Maximum (LGM) and the early Late glacial (25,000–14,700 years ago) in Iberia (lineage A), the Balkan region (lineage C) and the Mediterranean (lineage B) [3, 5, 6]. Lineage B has originally been attributed to red deer from Sardinia/N-Africa, but has recently been associated, based on ancient DNA material from mainland Italy, to red deer from the Italian refugial lineage [7]. The existence of a fourth potential mtDNA lineage was proposed for the Middle East [8]. Following the LGM, the Iberian lineage A) colonized the British Isles and parts of Central Europe, while the eastern lineage (haplogroup C) from the Balkans colonized south-east central Europe and the Carpathians, reaching as far north as Austria, Hungary and the Czech Republic. In addition, northern areas may have acted as cryptic refugia in North-West Europe through the LGM [9].The Bavarian-Bohemian region was identified as part of a suture zone between western and eastern European red deer matrilines [10]. However, a detailed sampling for molecular analyses is missing from other regions of Central Europe, including the region from the North of the Swiss Alps to the East of France [3, 4, 5, 9].

As a major game species, red deer has been exposed to a variety of anthropogenic pressures across the whole of Europe. Hunting pressure and habitat alterations have been particularly strong during the last centuries, driving many populations to extinction or confinement in small areas. These anthropogenic influences have greatly influenced the genetic diversity and structure of red deer populations across Europe [11, 12, 13, 14, 15, 16].

Red deer slowly recovered after the Second World War, thanks to protective laws, hunting reserves and restocking with foreign specimens [11]. The increase of forest areas and the extinction of natural predators also facilitated a rapid population expansion, at least at a local level. Overall in France after the Second World War, the expansion of red deer has been highly variable, fluctuating in response to e.g., the local hunting laws or forestry demands. Mountainous areas have repeatedly acted as retreats of threatened native red deer populations as it was the case in the Vosges Mountains, where a few hundred (300–500) individuals survived in remote summits [17]. After the 1950s, the population expanded rapidly to an estimated population size of more than 20,000 relatively free ranging individuals in 2015–2016 (data compiled from results of shooting, published by the directions of territories of the Vosges).

Facing the increase of browsing damage, modern forestry calls for a strong reduction of red deer densities, arguing that the species originally lived in grassy landscapes and are not indigenous to the forest [18]. Red deer is, however, known to be an opportunistic mixed feeder, adapted ethologically or ecologically to a large range of habitats [19, 20, 21], and recognized as a central element of forest functioning, for its positive impact on vegetational composition and structure if also exposed to predation risks [22, 23, 24].

In this study we aimed to reconstruct the history of north-eastern France red deer populations, using ancient and contemporary mitochondrial DNA (mtDNA) sequences covering the last 7’000 years. More precisely, our goals were (i) to explore the maternal lineage(s) of the red deer population in this region through time; (ii) to identify changes in spatial and temporal structures, and the genetic (haplotype and nucleotide) diversity; and (iii) to analyse the feeding habits, through the inspection of carbon and nitrogen stable isotope ratios in archaeological samples to understand ungulate-vegetation correlations throughout millennia in the Vosges and the adjacent plains. Indeed Plants growing under the dense, closed canopy are known to exhibit lower ^13^C abundances compared to the same plant groups growing in anthropogenic landscapes, generally composed of mosaics of meadows and species-rich small woodlands. As ^13^C/^12^C ratios (expressed as δ^13^C values) measured on the collagen of large herbivores reflect aspects of their diet, it can be used for evaluating the degree of vegetation canopy closure (the so-called “canopy effect”) in the areas where they used to forage [25]. The carbon isotope signature can also be used to track the presence of dense forest through time [26, 27, 28]. Differences in δ^15^N values of bone collagen of large herbivores are also interesting because they reflect soil composition and altitudinal gradients where the consumed plants used to grow [29, 30]. In particular, red deer living at higher altitude in the Vosges massif are thus expected to exhibit lower δ^15^N than those dwelling at lower altitude. For instance, important variations in δ^13^C and δ^15^N values were described in red deer bone collagen from the Late glacial and the early Holocene in north-eastern France and neighbouring areas when the climate became warmer and vegetation cover denser e.g. [31, 32].

## The north-east of France: Natural context

### Geology and soil characteristics

**Fig 1:**
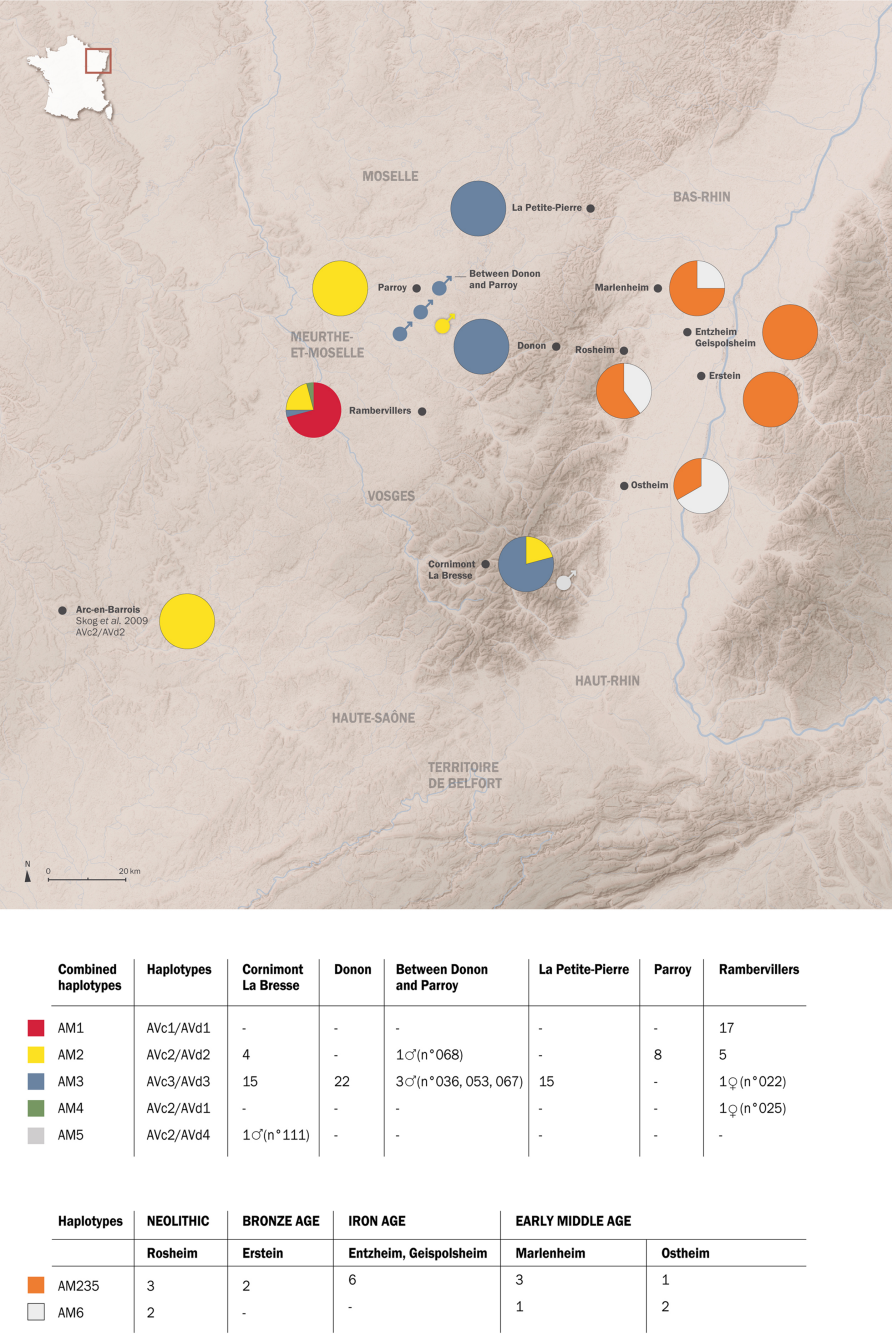
Modern and archaeological study sites and distribution of ancient and contemporary red deer mtDNA haplotypes in north-eastern France. Pie charts on the right side show the relative distributions of ancient haplotypes AM235 and AM6, based on concatenated cyt b and d-loop 256 bp. Pie charts on the left side show the relative distribution of modern haplotypes (AM1 to AM5) based on combined 680 bp cyt b and 785 bp d-loop sequences. The sex of modern male samples is added when they are culled near or in a territory from which they are not originated. Drawing: Delphine Souan, Archéologie Alsace, France (Maps ASTER, Nasa).

The Vosges Mountains (47°40’ to 48°50’N, 6°40’E to 7°30’E) form a long ridge with a continuous crest line (a maximum elevation of 1424 m above sea level (asl.) at their highest point) (Fig 1). These mountains consist of a central area of magmatic rocks, such as granite and gneiss; sandstone covers the northern part of the mountain ranges. In the adjacent Rhine rift valley east of the Vosges, landscapes correspond to a variety of nested terraces of different ages, altitudes and soil characteristics (loess or alluvial sediments) depending on the local geology and river dynamics [33, 34, 35].

### Climate

Mean annual temperatures range from 9°C at 400 m (asl.) to 4°C at 1200 m asl. Depending on elevation, mean winter temperatures range between -6°C and -1°C, while mean summer temperatures range between 8°C to 14°C. The western windward slopes of the Vosges Mountains are characterised by an oceanic climate with 800–1000 mm precipitation annually, which increases to 2000 mm towards the crest. As a consequence of the crest effect, the climate is semi-continental with a mean annual temperature of 11°C and less than 700 mm precipitation annually in the Rhine rift valley.

### Vegetation

In the Vosges, the dominant forest types are beech fir forests (above 500 m asl.), beech/oak shady forests (above 350 m asl.). The lowland loessic terraces and Rhine riverine terraces (150 m asl) are dominated by thermophilous oak/ash/hornbeam forests. These lowland forests are naturally rich in herbaceous species in their understory, due to the properties of their canopies (i.e. light species and heterogeneous architecture, in particular in alluvial environment [36, 37]). Lowland forests were subjected to deforestation from the Neolithic times onwards [38]. As a result, landscapes of the Rhine plain and loessic terraces have been composed of a mixture of meadows and species-rich woodlands since millennia.

### The history of red deer

Red deer was regularly recorded at archaeozoological sites of the Rhine plain and adjacent hill foots from the Neolithic to the early Middle Ages [39, 40, 41, 42, 43, 44, 45] (S1 Text and S1 Table). The species was abundant during historical times until the beginning of the 18^th^ century, then declined after the French Revolution through overhunting [46, 47]. At the very end of the 19^th^ century, red deer populations nearly went extinct in France. German texts of the 1880s recorded the survival of a few hundred individuals in the Donon massif in the north of the Vosges [17]. Red deer survived also in a few private forests of adjacent plains such as Arc-en-Barrois, in the south-west of the Vosges, where hunting with hounds was practiced.

During the second part of the 20^th^ century, the red deer population suffered from human selection of large antlers and local habitat fragmentation [48, 49].

## Material and methods

### Ethics statement

This research did not involve purposeful killing of animals. Samples of modern red deer were gathered from dead free-ranging red deer legally shot by hunters during the depopulation management plan launched by the regional prefects. Therefore animals were not killed specifically for the study, and the sampling and the study of the gathered material (muscle and tongue tissues) did not require an additional approval of the ethics committee.

### Sampling

#### Modern samples

We extracted DNA of muscle or tongue tissues of 117 red deer individuals (for details see S2 Table; Fig 1) from 1. the Vosges mountains, namely the “Donon Massif” (48° 30’N; 7° 09’ 54 E, altitude: 1009 m), “La Petite-Pierre” (48° 51’N, 7° 19’E, altitude: 397 m), “Cornimont /La Bresse” (48° 00’ N, 6° 52’ E, altitude: 1363 m), 2. The Lorraine plateau, namely « Rambervillers” (48° 20’ N, 6° 38’E, altitude: 272 m) and the “Parroy massif” (48° 41’ N, 6° 36’ E, altitude: 303 m) 3. a transitional region of low altitude between the “Donon” and “Parroy” massifs (S3 Table; Fig 1). All animals were hunted between October 2013 and January 2014. Note that there is no physical or ecological barrier between all these sites.

#### Archaeological samples

We analysed mitochondrial DNA (mtDNA) as well as carbon and nitrogen stable isotopes (expressed as δ^13^C and δ^15^N values) of 23 archaeological bones from the following archaeological sites in Alsace region: Marlenheim (48° 37’N; 7°29’E, altitude: 270 m); Entzheim (48° 31’N, 7°37’ E, altitude: 153 m), Erstein (48° 25’N, 7°39’ E, altitude: 157 m), Rosheim (48° 29’N, 7°28’ E, altitude: 164 m), Ostheim (48° 09’N, 7°22’E, altitude: 186 m).

**Fig 2:**
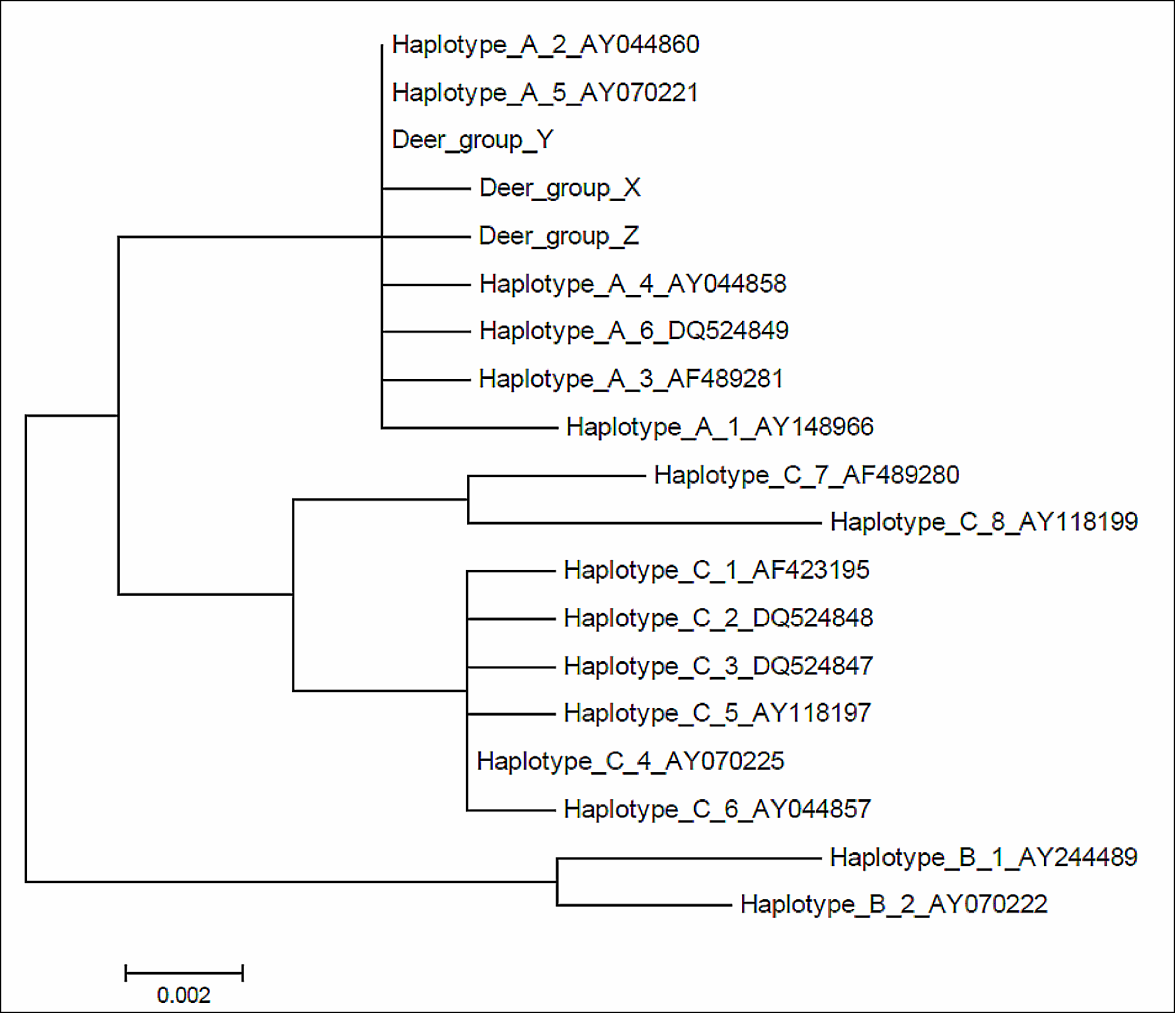
Haplotype genealogy based on maximum likelihood. Mitochondrial haplotype genealogy based on the mitochondrial control region and a maximum likelihood phylogenetic analysis. The haplotypes detected in red deer from the Vosges are indicated in red, all other haplotypes were taken from [3]. Haplotypes belonging to haplotype lineage A are indicated in black, those of haplotype lineages B, C are depicted in grey. Note that some of the haplotypes initially identified by Skog et al. 2009 have collapsed into a single haplotype in our analyses, which is due to the use of a shorter sequences alignment in our analysis to match the length of the newly obtained sequences.

Bones were dated by archaeological typology from the Neolithic (end of 6^th^ and beginning of 5^th^ millennium BC), the final Bronze Age (1026–1010 BC), the Iron Age (Hallstatt D3-La Tène A1, 475 BC), and the early Middle Age (600–750 AD) (S1 Text and S1 Table; Fig 2).

### Methods

#### Modern sample. Preparation, DNA extraction and PCR analysis

Modern samples were processed at two different laboratories in Bern and in Basel. These are different to the laboratories in which the historical samples were processed, in order to avoid cross-contamination. Additionally, genetic sexing of five modern samples was performed at the Leibniz Institute for Zoo- and Wildlife Research (IZW), Berlin, Germany. The details of the protocols have been performed independently and are, hence, independently reported in the following sections.At the Institute of Genetics, University of Bern, processing was as follows: DNA was isolated from thin and small pieces (~0.5 cm^3^) of tongue or muscle tissue of 24 modern red deer samples (DEER 094–117) by using the Nucleon Bacc2 kit following the manufacturer’s instructions. The cytochrome b region (cyt b) was amplified using the primers cerni cytoB A1: GAAAAACCATCGTTGTCATTCA [7] and cerni cytoB B2a: GGAGGTTRGTAGCTCTCCTTTT (modified [7]) and the AmpliTaq Gold 360 Master Mix (Applied Biosystems) with the following conditions: 20 μl volume containing 1 μl of each primer (final concentration 0.5 μM), 10 μl ATG Mastermix (AmpliTaq Gold 360 Master Mix), 2 μl of 360 GC enhancer, 4 μl of H_2_O, and 2 μl of 10-fold diluted template DNA. PCR amplifications were modified according to [50]: starting with a denaturation step at 95°C for 10 min followed by 35 reaction cycles (denaturing at 95°C for 45 s, annealing at 54°C for 45 s, and extension at 72°C for 70 s) and final extension for 10 min at 72°C. Following treatment with shrimp alkaline phosphatase (Roche) and exonuclease I (New England Biolabs) PCR products were Sanger sequenced by Microsynth (Balgach, Switzerland) using PCR primers as sequencing primers (in both directions).

At the Zoological Institute, University of Basel, processing was as follows: a fraction (~0.5 cm^3^) of anonymous tongue or muscle tissue of 93 samples (DEER 001-DEER 093) was digested using Proteinase *K* and the total DNA was obtained by means of a high-salt extraction method [7]. DNA concentration was assessed for each sample using a NanoDrop spectrometer (NanoDrop Products, Thermo Scientific). The mtDNA sequence of the cytochrome b region (cyt b) was amplified over a length of 680 bp (forward sequence) using the previously published primers cerni cytoB A1 and cerni cytoB B2 [7] on a Veriti cycler (Applied Biosystem) using REDTaq DNA polymerase (Sigma-Aldrich). PCR amplification was performed following [7]. First, a denaturation step at 94°C for 3 min was performed, followed by 35 reaction cycles (denaturing at 94°C for 45 s, annealing at 54°C for 45 s, and extension at 72°C for 70 s) and concluding with a final extension step of 3 min at 72°C. PCR fragments were purified using ExoSAP-IT (USB, Thermo Fisher Scientific). The PCR fragments were subsequently sequenced with the BigDye sequencing chemistry (Applied Biosystems) and analyzed on a genetic analyzer (ABI 3130 *xl*, Applied Biosystems). The cyt b sequence of 105 out of the total 117 red deer samples was successfully determined (for details see S2 Table). A partial sequence (785 bp) of the mitochondrial control region (D-loop) was determined for 99 individuals, following the PCR protocol of [49] and using primers CE-CR-FOR and CE-CR-REV [51] (for details see S3 Table). In total for 93 individuals sequences from both markers were available.

DNA sequences were inspected by eye in CodoneCode Aligner (version 3.5.6, CodonCode Corporation) and, when available, forward and reverse sequence reads were assembled. Sequence alignment was done with ClustalW as implemented in the software MEGA (version 6.06: [52]). Identical sequences were collapsed into mitochondrial haplotypes based on an initial round of phylogenetic analyses with MEGA. The so identified control region haplotypes were combined with the available sequence data from [3], and trimmed to the sequence length used by these authors. We then established a haplotyped genealogy following the method described in [53] on the basis of a maximum likelihood tree obtained with PAUP* [54] and the GTR + I model of molecular evolution (based on a model selection test implemented in MEGA). This analysis was only performed for the control region haplotypes, as many more sequences covering a larger geographic area are available on GenBank for this marker compared to cyt b. Modern red deer sequences are available at GenBank under accession numbers: MF177755-MF177853.

At the Leibniz Institute for Zoo- and Wildlife Research (IZW), Berlin, Germany, processing of genetic sexing was as follows: data about sex were only available for 45 red deer samples, and some critical samples lack this information. Indeed knowledge of the sex is important for evaluating the possible persistence of haplotypes in a given population, only possible by the presence of females. We have thus completed this information by selecting five samples, which were genetically sexed using the zfx/zfy loci according to [55].

### Archaeological samples. Preparation, DNA extraction and PCR analysis

The 23 ancient red deer samples were processed at IPAS (Basel, Switzerland), following accepted standards in a DNA research [56, 57] established at IPAS, e.g. [58]. Sample preparations including DNA extractions and pre-PCR steps were performed in dedicated a DNA facility that was physically separated from post-PCR laboratories.

A few millimetres of the outer surface of red deer bones were removed with sand paper and bone powder was obtained by drilling into the sanded area at moderate speed using a Dremel tool. Silica-based DNA extractions followed the User-Developed Protocol: “Purification of total DNA from compact animal bone using the DNeasy^®^ Blood & Tissue Kit” (Qiagen, Basel, Switzerland) for less than 100 mg. Every four to five samples one mock extraction was performed. The yield was about 150–200 μl per sample after purification of extracts with AE buffer (provided from the Kit) on 30 kDa centrifugal filters (Amicon/Millipore, Zug, Switzerland). Extracts were stored at -20°C.

Two mtDNA fragments, one within the cytochrome b (cyt b) gene (168 bp) and one within the mitochondrial d-loop (94 bp) that are located within a longer segment of cyt b (430 bp) and d-loop (328–333 bp) DNA, previously targeted by [9], were amplified. To obtain the cyt b fragment two primer pairs were used to generate two smaller overlapping PCR fragments: RD cyt b A forward (5’-GTTGTCATTCAACTACAAGAA-3’) and cyt b A2R reverse (5’-TCCTAGTAATGAGCCGAAATT-3’) (98 bp-fragment); cyt b forward (5’-AACAACGCATTTATTGACCTC-3’) and cyt b reverse (5’-TGCTGTTATTGTATCAGATGTAT-3’) (100 bp). Both fragments were concatenated to a single fragment of 168 bp (position 14’157–14’324 of reference sequence GenBank AB245427). The d-loop fragment (94 bp) was amplified by using primer pair CR 2 forward (5’-ATCAAGAACTTTATCAGTATTAAATT-3’) and CR 2aR reverse (5’-ATGTACTATAAGCGCATAAR-3’) (position 15492–15585 of reference sequence GenBank AB245427). All primers used were taken or modified from [9]. Finally, the concatenation of both marker fragments into a single trimmed fragment of 256 bp allowed for distinction of the three major European *Cervus elaphus* mitochondrial lineages A, B and C, diagnostic positions according to [3].

PCR amplifications were performed in a Mastercycler pro S (Eppendorf, Allschwil, Switzerland) in 25 μl volumes with 5–8 μl DNA sample, 2 μM each primer, 400 μM dNTP Mix (Promega, Dübendorf, Switzerland), 1.5 U AmpliTaq Gold, 1× GeneAmp PCR Gold buffer (150 mM Tris-HCl, 500 mM KCL, pH 8.0) and 2 mM MgCl_2_ (all from Applied Biosystems, Hombrechtikon, Switzerland). PCR started with a 12-min initial denaturing step, followed by 70 cycles of denaturing at 94°C for 1 min, annealing at 50°C– 52°C for 1 min, extension at 72°C for 1 min, with a final extension of 5 min at 72°C. Every amplification included non-template controls (at least one per four to five samples). For reliability and authentication of sequences, at least two PCR products from two independent extractions were analysed. Extract and PCR controls were without PCR product of the expected size. Because longer cyt b fragments of red deer (276 bp and 477 bp) could be generated repeatedly from three samples (CER9, CER17 and CER21, data not shown) indicative of modern DNA contamination, these samples were withdrawn from further analysis.

Amplicons were visualized on and gel purified from 3% agarose gel by using MinElute Gel Extraction Kit (Qiagen, Basel, Switzerland). Products were directly Sanger sequenced by Microsynth (Balgach, Switzerland) using the same PCR primers as used in amplification reactions, but to which a non-specific 40-bp nucleotide tail (5’-AACTGACTAAACTAGGTGCCACGTCGTGAAAGTCTGACAA-3’) had been added to the 5’-end [59].

Sequences were aligned with BioEdit [60] using the *Cervus elaphus* reference mitochondrial genome AB245427. Concatenated sequences of 256 bp generated in this work (20 ancient and 93 modern sequences; samples with missing data were excluded, see S3 Table) along with published ancient and modern red deer sequences representing the European range of the species [8] were used to build a median joining network [61] with NETWORK 5.001 using default weight for all characters except character 1 which was weighted with 50 [62]. Diversity measures nucleotide diversity (ND), and haplotype diversity (Ht div) were calculated with Arlequin 3.5.2.2 [63] using the concatenated 256 bp fragment. Ancient sequences were compared to modern red deer sequences from the five Vosges populations.

Ancient red deer sequences have accession numbers: GenBank: MF359564-MF359583.

#### Sample preparation and stable isotope analysis

For each ancient bone specimen, a small fragment was cut with a rotating tool equipped with a circular diamond-coated blade, ultrasonicated in acetone and water, rinsed with distilled water, dried and crushed to a powder of 0.7 mm grain size [64]. Then, an aliquot of around 5 mg was used to measure the nitrogen content (%N) of the whole bone, in order to screen out samples with excessive collagen loss [65, 66]. For instance, fresh bones contain around 4% nitrogen, while ancient bones with less than 0.4% nitrogen usually fail to yield good collagen, and bones with 0.4 to 1% may or may not yield good collagen depending on the context.

The measurements were performed using a Vario EL III elemental analyser using Sulfanilic acid from Merck as internal standard. The mean standard errors were lower than 0.05% for %N. The collagen was purified according to a well-established protocol by [66]. The elemental and isotopic measurements were performed at the Isotope Geochemistry working group of the Department of Geosciences at the University of Tübingen (Germany), using an elemental analyser NC 2500 connected to a Thermo Quest Delta+XL mass spectrometer. The elemental ratios C/N were calculated as atomic ratios. The isotopic ratios are expressed using the “δ” (delta) value as follows:

$$\delta^{13}C=[\frac{{{(}^{13}C{/}^{12}C)}_{sample}}{{{(}^{13}C{/}^{12}C)}_{reference}}-1]*1000\;\text{and}\;\delta^{15}N=[\frac{{{(}^{15}N{/}^{14}N)}_{sample}}{{{(}^{15}N{/}^{14}N)}_{reference}}-1]*1000\;\left(‰\right)$$

The international references are V-PDB for δ^13^C values, and atmospheric nitrogen (AIR) for δ^15^N values. Measurements were normalized to δ^13^C values of USGS24 (^13^C = -16.00‰) and to δ^15^N values of IAEA 305A (δ^15^N = 39.80‰). The reproducibility was ±0.1‰ for δ^13^C measurements and ±0.2‰ for δ^15^N measurements, based on multiple analyses of purified collagen from modern bones.

The reliability of the isotopic signatures of the extracted collagen was addressed using their chemical composition (%C, %N, and C/N ratios). These values must be similar to those of collagen extracted from fresh bone to be considered reliable for isotopic measurements and radiocarbon dating. Several studies have shown that collagen with atomic C/N ratios lower than 2.9 or higher than 3.6 is altered or contaminated, and should be discarded, as well as extracts with %N < 5% [67, 68].

Non-parametric Wilcoxon paired test was performed with software JMP 13.0

## Results

### Genetic composition and spatial distribution of modern red deer haplotypes

#### Modern red deer mtDNA haplotype analysis

In total, we obtained mtDNA sequences for most of the 117 modern samples (105 in cyt b and 99 in the control region). All sequences clustered into the western European red deer haplotype A (see haplotype genealogy based on 785 bp of the control region sequences in Fig 2).

**Fig 3:**
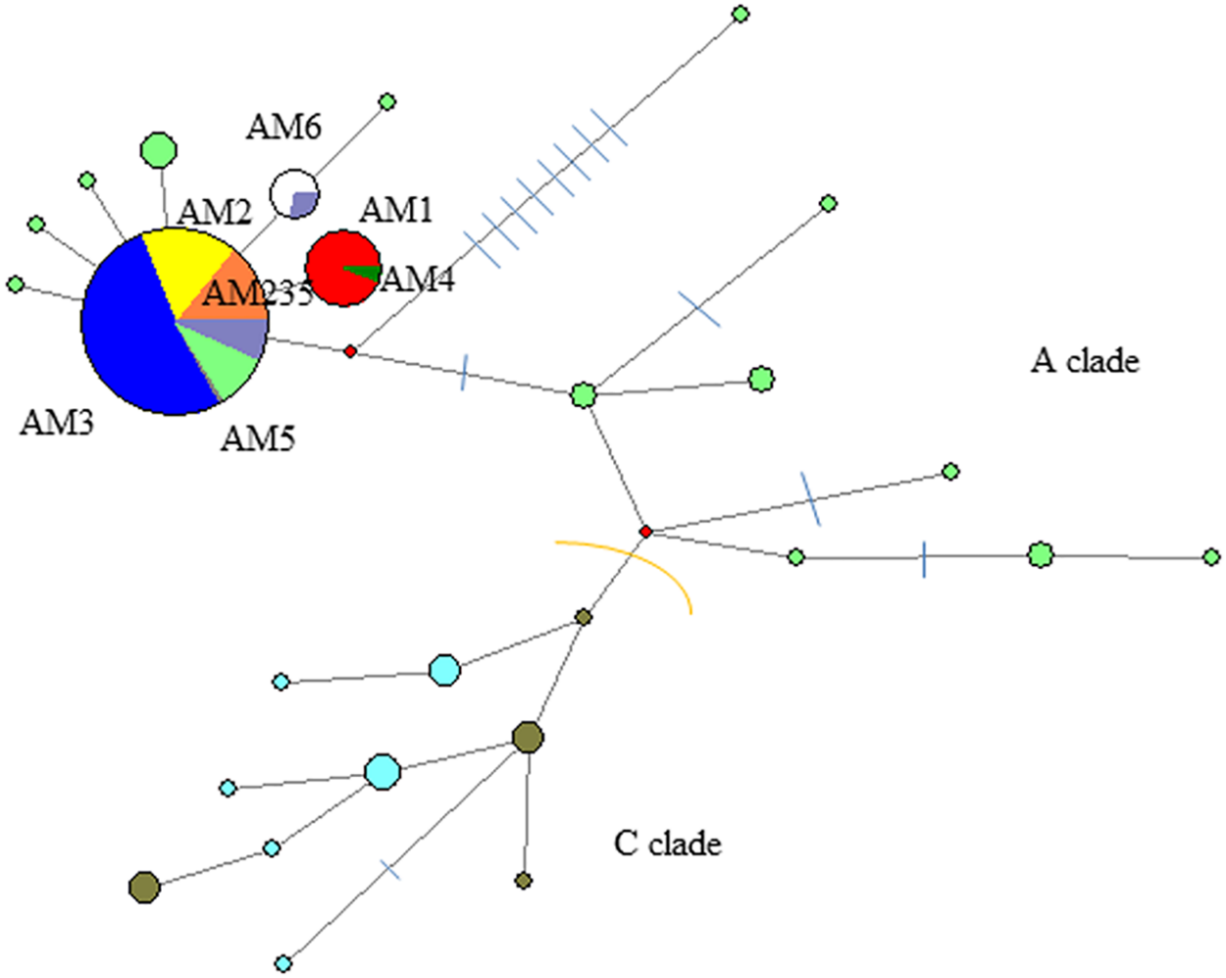
Median joining network of combined cyt b and d-loop sequences (256 bp) showing lineage affiliations of ancient and modern red deer from the Vosges compared to published ancient and modern sequences from the European range of red deer. The networks includes ancient red deer (haplotypes of the A clade from the Vosges (AM235 = orange; AM6 = white), modern red deer from the Vosges (red = AM1, yellow = AM2, dark blue = AM3, green = AM4, grey = AM5), published western-central European ancient and modern haplotypes from the A and C clades (modern A clade = grey blue, modern C clade = olive, ancient A clade = light green, ancient C clade = light blue; data from [10]. The size of the circles are proportional to the number of individuals, dashes indicate mutational steps.

The cyt b sequences of the modern Vosges red deer samples grouped into three distinct mtDNA haplotypes, tentatively named AVc1 (n = 18), AVc2 (n = 23) and AVc3 (n = 64); four haplotypes were found in the d-loop, tentatively named AVd1 (n = 19), AVd2 (n = 19), AVd3 (n = 60) and AVd4 (n = 1)(data not shown). As both markers from the same individuals were sequenced, we also performed an analysis with the two mtDNA markers combined, resulting in five combined haplotypes which we tentatively named AM1 (AVc1/AVd1, n = 17; AVc1/unknown, n = 1; unknown/AVd1, n = 1); AM2 (AVc2/AVd2, n = 18; AcV2/unknown, n = 3; unknown/AVd2, n = 1); AM3 (AVc3/AVd3, n = 56; AVc3/unknown, n = 8; unknown/AVd3, n = 4); AM4 (AVc2/AVd1, n = 1); AM5 (AVc2/AVd4, n = 1) (S2 Table). When compared to available records in GenBank, AVc1 and AVd1 are unique, AVc2 and AVd2 are ubiquitous throughout western Europe (matching haplotype A3C (680 bp) and AD1 (332 bp), respectively, see Maximum likelihood tree Fig 3, and [3]). AVc3 and AVd3 match haplotypes A4C (680 bp) and AA9 (332 bp), respectively [3] previously reported only from France (i.e. La Petite-Pierre). Based on 331 bp of d-loop AVd4 showed 100% similarity to red deer sequences from Poland (HQ534304, HQ534309, HQ534310, KX496902, KX496903, KX496905, KX496915) [69, 70], and the Czech Republic (KM410131) [71].

#### Spatial distribution of maternal lineages

According to our analyses, the mtDNA haplotypes show strong spatial distribution (Fig 1). AM3 is widely distributed in the Vosges (a total of 56 individuals), with the highest number of animals in the “Donon” (22 individuals), followed by “Cornimont/La Bresse” (south) and “La Petite Pierre” (15 and 15 individuals, respectively). One female with haplotype AM3 was found in “Rambervillers”, and 3 males between the “Donon” and “Parroy”.

AM1 and AM2 were affiliated to 17 and 18 individuals, respectively. Red deer with AM1 were found only near “Rambervillers”, while AM2 was more widely distributed in the south of the Vosges. Interestingly, AM2 is absent in the “Donon” massif, except for one male individual (sample 068) hunted between the south of “Parroy” and the “Donon”.

AM4 and AM5 are both rare and located in the south of the studied area. In “Rambervillers”, the only found AM4 haplotype (sample 025) was a female. In “Cornimont/la Bresse”, only the male individual (sample 111) was identified as AM5 haplotype.

### Archaeological samples

#### Genetic composition of red deer from the Neolithic to early Medieval times

Cyt b and d-loop mtDNA fragments were reliably amplified and sequenced from 20 ancient samples (out of 23) coming from all periods tested, that are, the Neolithic, Bronze Age, Iron Age, and Early Middle Age periods (S1 Table).

Based on the 168 bp cyt b sequences all ancient red deer belong to the Western European haplogroup A and share the same haplotype. The 94 bp d-loop fragment was chosen to capture diversity and two haplotype groups were identified differing at position 15547 of the reference sequence AB245427. Sequences with the presence of nucleotide base Thymine “T” at that position (15547) are named AM235 to indicate identity to haplotypes AM2, 3 and 5 of modern red deer from the Vosges within the concatenated 256 bp fragment (GenBank MF359564-MF359583). The network shows that AM235 is frequent in ancient and modern Europe (Fig 3). Sequences with the presence of nucleotide base Cytosine “C” at that specific position are named AM6 to indicate a haplotype not found in our modern red deer dataset, and which is also very rare elsewhere today (Fig 3). It corresponds to three modern individuals from Eastern Europe (KF133877-KF133949, KF133875-KF133947, KF133878-KF133950.

Because of the presence of only two haplotypes defined by a mutation at one position, diversity measures are very low: Ht div = 0.39 ± 0.08, ND = 0.0015 ± 0.0016, and far below of Western / Central European archaeological red deer. There is zero diversity in the Bronze Age-Iron Age and diversity measures in Neolithic and Medieval times are similarly low. However, keeping in mind the low sample size, diversity might be higher. Compared to modern red deer of the Vosges, ancient diversity measures are slightly higher than the “Rambervillers” population, all other modern red deer population have zero diversity within 256 bp fragment (Diversity measures Table 1, Figs 1 and 3).

**Table 1:** Diversity measures (haplotype diversity = htdiv; nucleotide diversity = ND, mean number of pairwise distances = MNPD) within the concatenated 256 bp fragment of cyt b and d-loop in ancient and modern red deer from the Vosges in comparison with published ancient western-central European red deer [9].

Population	N	n Ht	ht div	ND	MNPD
Vosges ancient red deer all	20	2	0.3947 +/- 0.1006	0.001542 +/- 0.001675	0.394737 +/- 0.384043
Western-Central ancient red deer after LGM A clade	13	7	0.8333 +/- 0.0861	0.009916 +/- 0.006401	2.538462 +/- 1.457825
Vosges red deer Neolithic	5	2	0.6000 +/- 0.1753	0.002344 +/- 0.002567	0.600000 +/- 0.562226
Vosges red deer Bronze-Iron Age	8	1	0	0	0
Vosges red deer Medieval time	7	2	0.5714 +/- 0.1195	0.002232 +/- 0.002330	0.571429 +/- 0.520798
*Vosges modern red deer*					
Donon Massif	22	1	0	0	0
La Petite Pierre	15	1	0	0	0
Cornimont/Bresse	20	1	0	0	0
Parroy Massif	8	1	0	0	0
Between Donon and Parroy	4				
Rambervillers	24	2	0.4200 +/- 0.0824	0.001634 +/- 0.001715	0.420000 +/- 0.395580

Haplotypes AM235 and AM6 differ in their spatio-temporal distribution. While AM235 is ubiquitously present at all sites and time periods, AM6 was identified at only three sites (i.e Rosheim, Ostheim, Marlenheim, all situated close to the Vosges Mountains) and two time periods, the Neolithic and Early Medieval periods (Fig 1 and S1 Table).

#### Diet composition of red deer from the Neolithic to early Middle Age times

Nitrogen content in fossil bones ranged from 0.6 to 3.1% (S1 Table) indicating limited to very good collagen preservation. Based on previous negative experience with bones in a similar preservation stage, most specimens with less than 1% nitrogen content were not selected for collagen extraction. Therefore we selected only one of the three bones (i.e. CER16 and not 14 and 15) from the Marlenheim individuals, namely the one that had the highest nitrogen content. Altogether, collagen extraction was performed for eighteen bones, two of which yielded residues with too low % C and % N indicating diagenetic alteration (CER17 and CER23, both from the Rosheim site). Finally, the collagen isotopic values of sixteen red deer bones were used for palaeobiological investigations. For these samples, the δ^13^C values ranged from -23.9 to -20.8‰ and the δ^15^N values ranged from 3.2 to 8.6‰ (S1 Table), which is a large range for an herbivorous species. In sites where at least three specimens were analyzed (Entzheim-Geispolsheim, Ostheim and Rosheim), the δ^13^C and δ^15^N values exhibited large ranges, with δ^13^C values crossing the threshold value of -22‰ taken as an indication of foraging under closed canopy.

Interestingly, the δ^13^C and δ^15^N values of red deer that belong to the two different haplotypes exhibited clear differences. The specimens belonging to haplotype AM235 had higher δ^13^C and δ^15^N values when compared to those belonging to haplotype AM6, the p-values being 0.061 and 0.0112 (Wilcoxon each pair) for δ^13^C and δ^15^N, respectively. The differences were therefore statistically significant for δ^15^N and with a trend for statistical significance for δ^13^C. In contrast, neither the δ^15^N values, nor the δ^13^C values differed significantly according to the chronological periods: none of the p-values was lower than 0.07 and 0.28 for δ^13^C and δ^15^N values respectively, when pairs were tested (Wilcoxon method; DF = 1 for both cases (D13C and d15N).

#### Analysis of the relationship between genetic differentiation and diet composition

**Fig 4:**
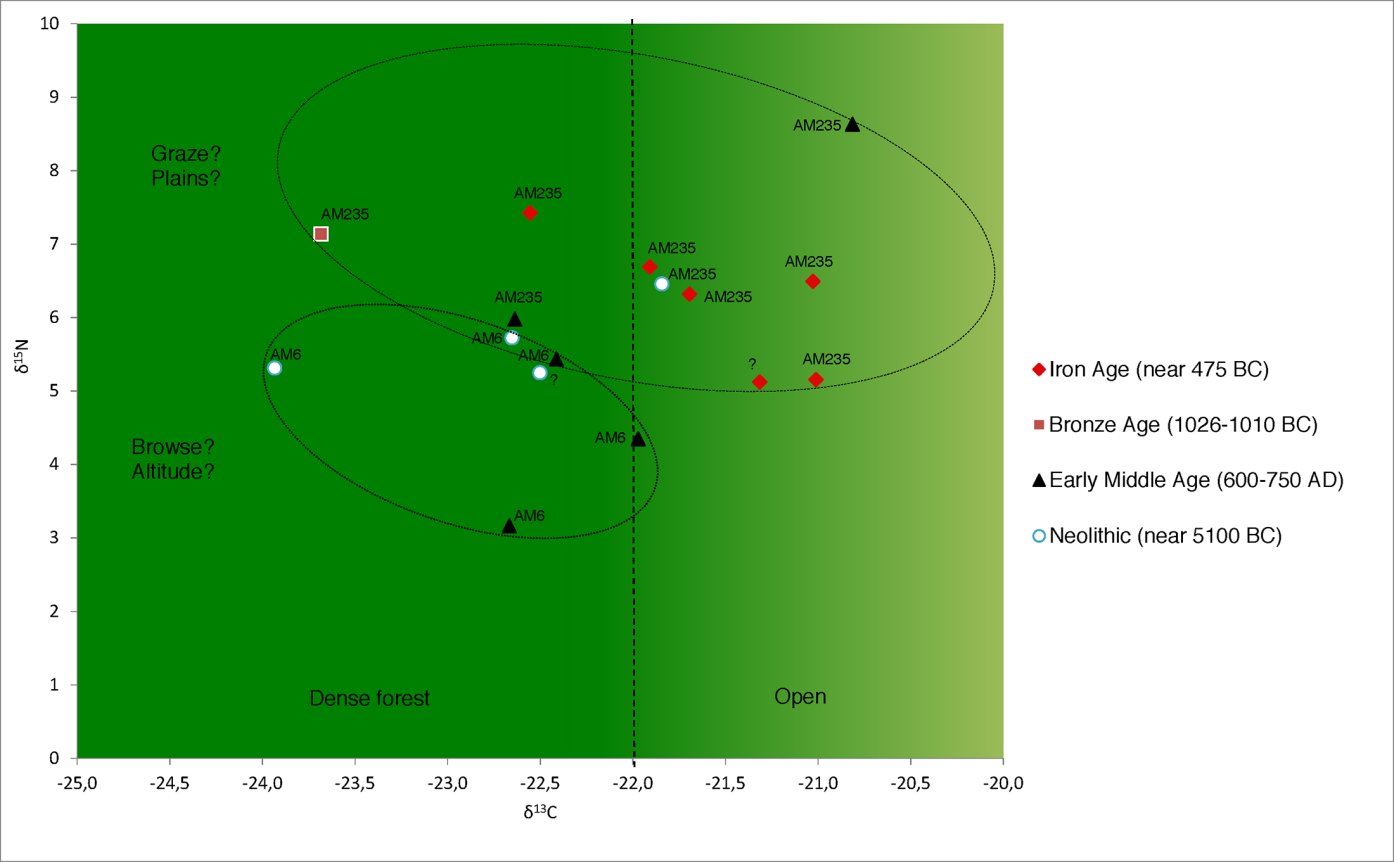
Scatter-plot of δ^13^C and δ^15^N values for archaeological red deer collagen according to their age and haplotype. Ellipses are drawn manually to encompass samples with the same haplotype. One ellipse (below) corresponds to individuals living deep in the forest (mostly browser habits); the other (above) individuals living in open habitats or open (plain) or herb-rich woodlands of the adjacent plains.

As shown in Fig 4, the AM6 haplotype seems to correlate with an “ecotype” that feeds preferentially in forested habitats while AM235 possibly correlates with an “ecotype” that select a variety of lowland habitats, from meadows to herb-rich woodlands.

## Discussion

Mitochondrial DNA cyt b and d-loop diversity patterns were studied in a range of regional red deer populations across Europe [1–16, 20]. Here we combine ancient and modern mtDNA d-loop data with isotopic data to infer temporal female lineage history, to compare historical sources with genetic data and to assess habitat exploitation in the past.

Our genetic analyses revealed that ancient and modern red deer samples belong to mtDNA haplogroup A suggesting that this haplogroup persisted during a period of at least 7000-years and suggest that the Vosges Mountains were re-colonized after the LGM from red deer derived from the Iberian refugium. In addition, our data suggest that no mixing, at least in the matriline, occurred with red deer population from other refugial origins. Since we did not detect lineage C haplotypes in that area (neither in the modern nor in the archaeological samples), the suture zone between these two groups is rather not located in north eastern France. Our choice of markers cannot exclude the possibility of mixing between local red deer stocks on a shorter temporal scale, including hybridization between populations from cryptic refugia.

Interestingly, only two and five mtDNA haplotype groups were present in ancient and modern samples of red deer, respectively, in the Vosges Mountains, both belonging to haplogroup/lineage A. This represents only a small fraction of the broad diversity of haplogroup A maternal lineages observed from archaeological samples before and after the LGM in western Europe [9, 15], or for example from Scotland [72] and Iberia [73]. However, due to the limited number of ancient samples, ancient diversity may be underestimated.

Possible explanations for this observation are a) the present day population has been re-colonized from a limited number of individuals maternal lineages from the Iberian refugium, also (perhaps) local refugia populations in the Vosges, combined with a loss of haplotypes through time [13] and b) the source population was large and genetically diverse but bottlenecks have occurred several times in the past, as shown in [40, 74]. According to the present state of knowledge of red deer history, we are not in the position to determine which of these two scenarios is more likely.

Regarding a combined interpretation of the isotopic and genetic analyses, the results further hint towards a tentative correlation between dietary (e.g. habitat type and the associated composition of plants) and genetic differentiation, with a potential for a “canopy effect” leading herbivores in forest mountain habitat (i.e. AM6) to have different isotopic values than those in lowland mixed landscapes (i.e. AM235). AM6 shows also a more restrictive habitat range than AM235, with a more specialized diet regime confined to forests of altitude.

Interestingly, no obvious ecological barrier has been identified that would prevent the free movement between the mountains and adjacent plains. It is thus conceivable that the driving force that may have led to the observed haplotype patterns is ethological. In polygynous species such as red deer mating is nonrandom because females are philopatric, while males tend to disperse. In red deer, females aggregate in matrilineal groups, promoting a social learning in offspring with respect to how and where exploiting familiar plant resources [75, 76, 77, 78]. These matrilineal groups probably also interbred [79, 80]. Similar conclusions suggesting that ecological features can constrain gene flow, in relation with habitat preferences, were published for other mammals in the world (e.g. [81, 82]). The diet differentiation however does not mean that populations with AM235 and AM6 haplotypes did not interact. Mountain populations of red deer usually migrate towards low altitudes during winter, which happens, however, outside the period of reproduction [83]. We suggest here that a nuclear DNA analysis could shed light on this issue.

A novel result is the persistence (several millennia) of geographically isolated populations of red deer in the Vosges Mountains. With regard to the ancient haplotypes, it might be speculated that such habitat partitioning may have lasted until the extinction of AM6, possibly perhaps too specialized and thus less adaptable to the changing environments after 1000 AD (among possible changes, let us quote Little Ice Age or increasing impact of humans on the natural world) or, alternatively, much later, during fauna overhunting after the French revolution. Another hypothesis would be the possibility of fungal, viral or bacterial infections to which specialized populations are particularly susceptible. This would illustrate the importance of genetic factors in clinical manifestations, and their influence on evolution [84, 85, 86].

The modern haplotype structure in the Vosges can largely be explained by historical events: Haplotype AM3 found shelter in the “Donon” and probably expanded as soon as overhunting stopped, which could explain its wide distribution in the north and the south of the Vosges. The relative frequency and relatively wide distribution of AM2 in the South of the Vosges Mountains suggest that there might have been a second refugium, other than the one in “Donon” massif, during the period of overhunting of the 19^th^ century. With regard to [3] we assume that this native haplotype has persisted in the former private hunting forest of “Arc-en-Barrois”, known since 1900 (François Klein, personal communication). It is possible that from this artificial refugium, red deer has expanded towards “Parroy” (at a distance of 150 km) and to the South of the Vosges (“Cornimont/La Bresse”; “Rambervillers”). This expansion possibly extended towards the South, as far from the “Donon” massif, where red deer had been exterminated [17]. That northward extension of haplotype AM2 was possibly prevented by the presence of local haplotype AM3.

Overall, our results hint towards the situation that behavioral difference associated with habitat choice, have influenced historical and present day population structure in red deer in Central Europe [72, 73]. While haplotypes AM1 and AM4 are still present in modern populations yet restricted to the south of the Vosges near “Rambervillers”. AM1 and AM4 haplotypes correspond to novel haplotypes from eastern geographic areas. AM1 in particular was favoured by successive owners from the hunting “Domaine de la Verrerie”, close to “Rambervillers”. The proportion of this haplotype in the sampling reveals that this population has been well established here. The lower frequency of AM4 can be explained by a selection by hunters. However as the individual AM4 killed here (025) was a female, this haplotype is also established in this adjacent part of the Vosges. Nevertheless the “Rambervillers” red deer population appears rather artificial. The probability of any presence of ancient AM5 haplotype is very low, because it was not observed in modern red deer in the Vosges. Indeed male 111 (haplotype AM5) found in “Cornimont/la Bresse” shows 100% similarity with red deer sequences from Poland (HQ534304, HQ534309, HQ534310) (GENBANK) based on its AVd4 haplotype. Many Polish individuals were kept in numerous small enclosures of the Vosges, from which male 111 has escaped (François Klein, personal communication). As it was a male, this haplotype was not established there.

AM3 is rare in this region compared to the north: only one female (022) was found. As for AM4, this indicates the presence of a small established population.

In conclusion, this study provides phylogeographical and ecological insights on the red deer population through time in North-eastern France. More precisely, we found that 1. despite heavy reduction in population size due to over-hunting in the last centuries red deer managed to survive and persist in the Vosges, with haplogroup A continuing to be present since Neolithic times. 2. Red deer lived on the very long term in a large range of forest habitats, from dense shady mountain forests to more open lowland forest habitats, thus exploiting a large variety of food sources. We thus suggest that these red deer populations should be protected against artificial selection or translocation in order to avoid loss of presumably populations. Future management strategies should avoid the elimination of lowland populations for increasing timber production. Together with reasonable hunting laws, more natural forestry practices and the return of a viable density of predators, these measures would allow the maintenance of red deer at reasonable densities in its whole habitat range.

## Supporting information

S1 Text Characteristics of archaeological sites yielding red deer samples. (PDF)

S1 Table Sample information, genetic data and δ13C and δ15N isotope profiles (%) for 23 subfossil bones of red deer analyzed in this study. Values in italics (CER17, CER 23) correspond to collagen with chemical composition outside the reliability range, therefore the δ13C and δ15N values are not considered for palaeobiological implications.
(PDF)

S2 Table Location and genetic data for 117 modern samples of red deer analyzed in this study. (PDF)

S3 Table Accession and extract numbers of red deer sequences from Meiri et al. 2013 used for diversity calculation (Arlequin) and network construction (Network 5.001).
(PDF)
