# Correction: Genetic diversity, genetic structure and diet of ancient and contemporary red deer (*Cervus elaphus* L.) from north-eastern France

**DOI:** 10.1371/journal.pone.0199945

**Published:** 2018-06-25

**Authors:** Annik Schnitzler, José Granado, Olivier Putelat, Rose-Marie Arbogast, Dorothée Drucker, Anna Eberhard, Anja Schmutz, Yuri Klaefiger, Gérard Lang, Walter Salzburger, Joerg Schibler, Angela Schlumbaum, Hervé Bocherens

Fig 2 is incorrect. Please see the corrected Fig 2 here.

**Fig 2:**
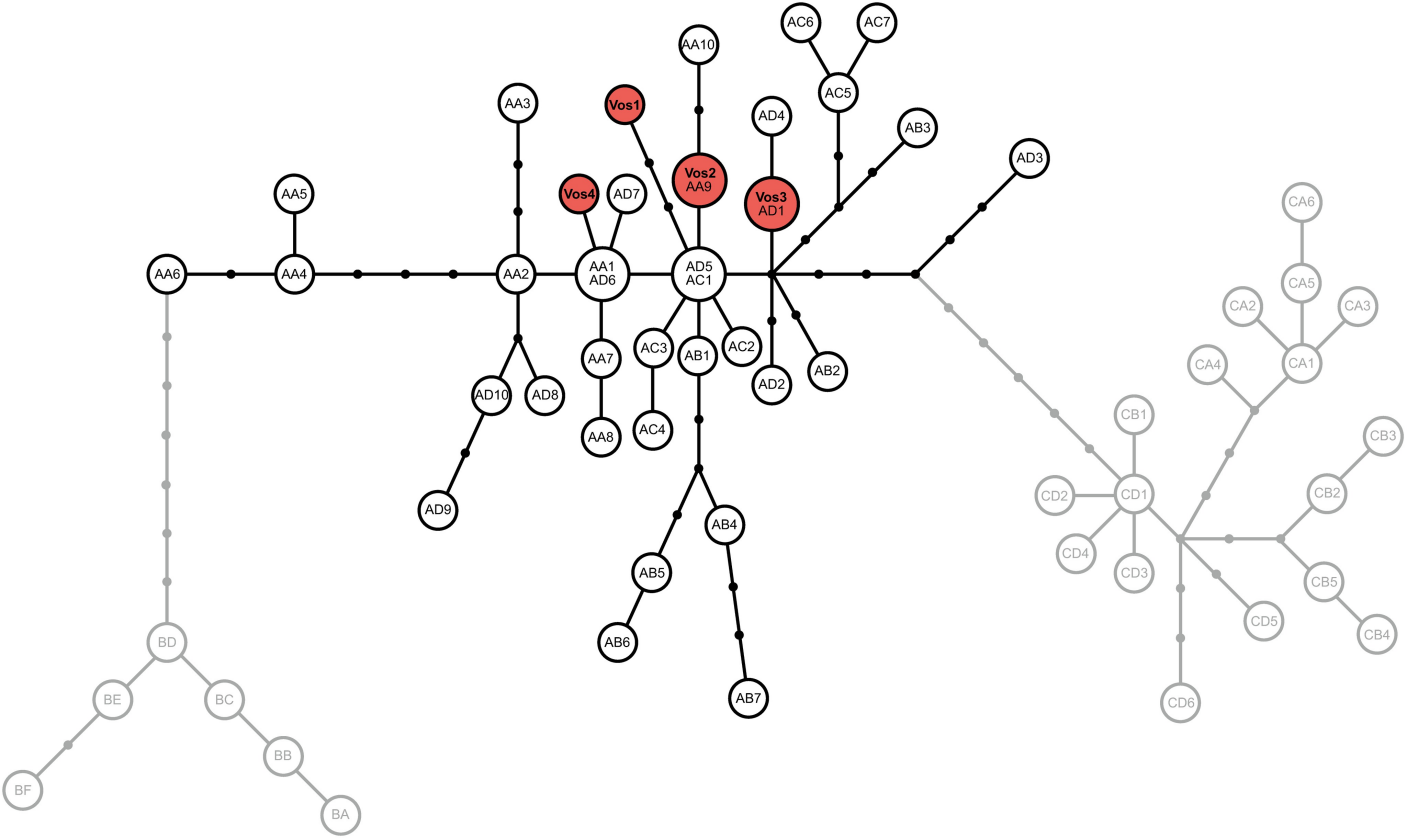
Haplotype genealogy based on maximum likelihood. Mitochondrial haplotype genealogy based on the mitochondrial control region and a maximum likelihood phylogenetic analysis. The haplotypes detected in red deer from the Vosges are indicated in red, all other haplotypes were taken from [3]. Haplotypes belonging to haplotype lineage A are indicated in black, those of haplotype lineages B, C are depicted in grey. Note that some of the haplotypes initially identified by Skog et al. 2009 have collapsed into a single haplotype in our analyses, which is due to the use of a shorter sequences alignment in our analysis to match the length of the newly obtained sequences.
