# Supplementary material for: Genetic diversity, genetic structure and diet of ancient and contemporary red deer (Cervus elaphus L.) from north-eastern France
Source: PLoS One. 2018 Jan 5;13(1):e0189278. doi: 10.1371/journal.pone.0189278 (PMC5755736; doi:10.1371/journal.pone.0189278)
Supplement: S1 Text — (PDF) [file pone.0189278.s001.pdf]

Supplementary file 1

Rosheim (France, Bas-Rhin)
The bone remains from Neolithic stags are from two sites, “Lotissement Sainte Odile” and “Laser”, in the Rosheim municipality (Bas-Rhin department, France). Both sites had settled on the loess sediment of the Alsace plain, close to the first hills of the Vosges and the Bischenberg massif. These two sites correspond to village-type occupations dated between the end of 6th and beginning of 5th millennium BC.

The “Lotissement Sainte Odile” Rosheim site was excavated in 1990 and has been allocated to Linear Pottery culture. It was mainly known for the discovery of an ancient Neolithic village and of a peculiar structure (Structure n° 108). This structure corresponds to a pseudo-ditch as it is composed of a series of aligned pits which were dug according to a precise plan and have been dubbed “Rosheim-type enclosure” [1]. The function of such an installation for which numerous analogs are described on early and middle Neolithic sites, has been the subject of intense debate. The diverse shapes and variable depths of the pits of which some were being dug while others were already abandoned suggest that their development proceeded over a long period and involved different groups. This rules out a defensive or an economic function and is more in favor of a ceremonial role. Among the bones that were selected in this study, 4 were found in the backfill of two pits (n° 13 and 19) of the pseudo-ditch, while two others come from a structure associated with habitats on the same site [2]. Seven red deer bones are studied in the present paper. Genetic composition and isotope profiles are able to individualize CER17, CER 18 and CER 20.

The Rosheim “Laser” site which was excavated in 1999 corresponds to a middle Neolithic occupation (Grossgartach culture) [3]. The most abundant remains were found in large ditches of loess extraction, which were subsequently filled with the daily waste from village activities. These included numerous animal remains of which we selected our stag bone from structure n° 122.

All analyzed stag bones from both sites share characteristic of consumed animals. They are isolated, dislocated and fragmented. Their presence in backfills associated with settlements indicates that stags were hunted and contributed to feed early and middle Neolithic populations. Stags were among the most hunted game of the time although most of the meat consumption was provided by the exploitation of domestic animals [4].

Erstein (France, Bas-Rhin)
The archaeological site is located on a loessic terrace close to the Rhine alluvial forest, Alsace plain, north eastern France close to the Erstein municipality. The building of areas of activity in the locality “Grassweg” led to an archeological monitoring on 30 ha. Within this zone, an archeological field work of 3.7 ha has been monitoring in 2007 by the Pôle d'Archéologie Interdépartemental Rhénan (PAIR) [5]. Human occupancy was continuous from 2231-2215 to the second half of the 10th century BC. Anthracology studies suggests the proximity of oak forests, but also transitional zones between forests and cultivated areas, for the transition from the Final Neolithic Bell Beaker to the Early Bronze Age (i.e.2200 BC) [6]. Carpologic data indicate also the presence of cultivated species and associated weeds [7]. Pits used to store grain, dating to the Final Bronze Age (1026-1010 BC) confirms the hypothesis of rather open landscapes in this loessic terrace at these periods [5, 8].
The chronological sequence (four phases recorded) dating to the Bronze Age has provided 2153 skeletal remains (25 kg, [9]). In the last phase (identified by dendrochrology of some fossil

woods to be aged of 1026 and 1010 BC), the number of archaeological bones was 814, whose 333 have been identified. All correspond to food wastes [10].
The pit 3008 provided an unusual quantity of wild preys: *Cervus elaphus* (N= 50, antlers excluded), *Sus scrofa* (N=4), *Ursus arctos* (N=1), *Bison bonasus* (N=1). Red deer was thus the most abundant prey for humans. Analysis of teeth allowed to distinguish four deer, where the analysis of tooth wear allowed to estimate the slaughter age: 3-4 years (1), 8-10 years (2), 10-12 years (1). The two red deer bones studied in the present paper, CER1 (age unknown) and CER2 (more than 36 M) belongs to this set.

Entzheim-Geispolsheim (France, Bas-Rhin)
This archaeological site is located in an alluvial terrace close to a paleochannel of a Rhine tributary (Bruche river), Alsace plain, north eastern France close to the municipality of Entzheim and Geispolsheim. The building of areas of activity in the localities “In der Klamm” and “Schwobenfeld” led to an archeological monitoring on about 23 ha. Within this zone, an archeological field work of 7 ha has been monitoring in 2006-2008 by the Pôle d'Archéologie Interdépartemental Rhénan (PAIR) [11, 12]. Human occupancy was continuous from the Neolithics to Early Middle Ages, except during the end of La Tène (1st century BC). The geomorphological study indicates an increasing human printing in the Bruche valley through deforestation and resource development as soon as the Neolithics [13]. Carpologic studies have revealed the presence of cereal crops and associated weeds, as well as forests and meadows since the end of the Bronze Age [14, 15]. An intense human impact was hypothesized for the Iron Age [11, 12], with regard to the number of silos found in the site around 475 BC (32 during the Hallstatt D3-la Tène A1), and around 440-375 BC (205 during La Tène A2-B1a). These data thus indicate a rather open environment as soon as the Protohistory.
23 000 skeletal remains, totalizing 332 kg [16, 17] were recorded for the whole protohistorical and historical periods. The seven bones of red deer studied in the present paper were all found in one chronological phase of the Iron Age (Hallstatt D3 – La Tène A1, near 475 BC). The total number of remains corresponding to this period was 2017 (18 kg), whose 725 were identified. All originated from discharges of food wastes (n: 1993). Most identified remains correspond to domestic animals (85%), although the products from hunting (15%) was higher than in other sites of the region. Wild animals consumed were diversified: *Cervus elaphus* (N=77), *Lepus europaeus* (N=9), *Sus scrofa* (N=6), *Capreolus capreolus* (N=6), *Felix sylvestris* (N=6), *Lutra lutra* (N=1), *Grus grus* (N=1), unidentified carnivore (N=1). The red deer appears to be the most consumed wild species (i.e. 11% of the total number of remains, antlers excluded).
In our sample, CER6 (age unknown), CER7 (about 30 M), CER8 (age unknown), CER9 (about 18 M), CER10 (more than 48 M), CER11 (about 30 M), CER12 (about 30 M), osteology and isotope profiles are able to individualize CER9, CER10, CER12.

Marlenheim (France, Bas-Rhin).
The archaeological site is located on a hill close to the Vosges (Kochersberg, Alsace plain, north eastern France (latitude, longitude, 187m) close to the Marlenheim city municipality. The archaeological monitoring on 17 000 m² was conducted in 2007-2008 by two organisms (PAIR, INRAP) in the locality ‘Hofstatt” [18]. Neolithic and protohistoric occupancy was detected in the site. The Merovingian period was documented by a housing estate and an artisanal unit. Carpologic data has suggested a diversified agriculture during early Middle Ages, mainly cereal crop, and fallows, but without pasture or meadow [19]. 10600 skeletal remains were discovered in the “Hofstatt” locality [20]. The study of 1568 remains (19.6 kg) from one structure aged of 600-650 AD, shows the prevalence of domestic animals among the 853 remains identified. The wild fauna is rare (less than 1%): *Sus scrofa* (N=1), *Cervus elaphus* (N=6) including a female aged 30-48 months. The four samples of red deer studied in our paper belong to this early

Middle Ages period. Genetic composition differentiated CER13 (age unknown) *versus* CER14-15-16 (three bones for a same doe, 30-48 M old).

Ostheim (France, Haut-Rhin).

The archaeological site is located between the hills close to the Vosges and a large wetland close to the Rhine (180 m; latitude, longitude) near the Ostheim city municipality. The building of areas of artisanal activity in the locality "Birgelsgaerten" led to an archeological monitoring on 9000 m², carried out ty the PAIR in 2008 [21]. The excavation has revealed a first occupancy during the Middle of the Neolithics, and a second during the beginning of La Tène. A rural settlement dating to the early Middle Ages, is sequenced into five occupation phases, from the 6th-7th century to the 9th-10th century AD. This habitat was situated on the upper part of alluvial cones of two confluent rivers [21].

Palynological studies were carried out within the sediments of a paleochannel which crossed the rural site [22]. They show that during early Middle Ages the landscapes consisted of mosaics of humid meadows, cultivated areas, and wetland forests of alder, willow and oak, typical of this region [23]. Other information come from anthracological studies, which include the human occupancy from the beginning of the Iron Age (Hallstatt D) to the third phase of the early Middle Ages (7th-8th AD). From these results we know that species tree used come from oak-hornbeam, oak-beech lowland forests or fallows, but also beech-fir forests from the Vosges. It seems also that early Middle Age landscapes were more open than other lowland sites of the region at the same period.

Carpologic data of food waste corresponded mainly to the phase 3 of early Middle Ages (7th-8th AD) [24]. They show the dominance of crop and legumes and associated weeds. Some wild plants come from wetlands or forest edges.

3500 skeletal remains (98 kg) were collected [25]. Most of them (i.e. 2480 remains with 1218 determined) are food wastes from early Middle Ages [20, 25].

An isotopic analysis of the remains of large preys [26]. showed that bovids, elk and red deer lived in dense forests, while one doe lived in a more open landscape, perhaps used as a bait [20, 26]. The importance of large game could be attributed to hunting activities of the local aristocracy [20]. Several other indices (i.e. low number of written sources for this site [26], the proximity of a wetland [27], the localization of Osthem within a set of royal and ducal early Middle Ages domains and situated half way between the Rhine floodplain and the Vosges), suggest that this site could be a game reserve without barriers [20].

In the pit 3070-3325 (650-750 AD), wild species totalized 81 remains, and are dominated by large game (N=50): *Cervus elaphus* (N=25), *Bison bonasus* (N=5, 3 animals), *Alces alces* (N=3), *Sus scrofa* (N=8), *Lutra lutra* (N=1) and different bird species (N=30). This indicates a clear contribution of hunting practices to food habits. Among red deer samples, we found 4 different individuals, including a young male of 12-30 months, and 3 others aged of more than 30 months (including one doe). The three red deer samples studied in the present paper, CER3, CER4, CER5 (more than 36 M), individualized by genetic composition and isotope profiles, come from this pit.

References

[1] Jeunesse C., Lefranc P. Rosheim „Sainte-Odile" (Bas-Rhin), un habitat rubané avec fossé d'enceinte – Première partie: les structures et la céramique. Cahiers de l'Association pour la Promotion de la Recherche Archéologique en Alsace, 1999, 15: 1-111.

[2] Arbogast RM. L'habitat rubané de Rosheim « Sainte Odile ». Etude de la faune. Cahiers de l'association Pour la Promotion de la recherche archéologique en Alsace. 16, 2001. p. 51-76.

[3] Lefranc P., Mauvilly M., Arbogast RM. L'habitat néolithique moyen (cultures de Grossgartach et de Roessen) de Rosheim « Laser » (Bas-Rhin). Cahiers Alsaciens Pour la Promotion de la Recherche Archéologique en Alsace, 15, 1999, p. 5-43.
[4]. Arbogast RM. La chasse au cerf au Néolithique. Un gibier par excellence. In : Chasses, Archeopages 28, 16-19, INRAP Paris; 2010.

[5] Croutsch C., Tegeroutsch C., Tegel W., Nicolas T., Pascutto E., Billot M., Leprovost C., Gratuze B., Logel T., Putelat O. Les sites protohistoriques d'Erstein « Grasweg-PAE » (Alsace, Bas-Rhin) : l'occupation Rhin - Suisse – France orientale. Revue Archéologique de l'Est 2011, 60 : 83-146.
[6] Nocus N. Forêts et Sociétés aux étages planitiaires et collinéens de l'Alsace du Néolithique au haut Moyen Âge : approche dendro-anthracologique. Thèse d'archéologie. Paris : Muséum National d'Histoire Naturelle, 2014, 363 p.
[7] Martinoli D. Analyse carpologique. In : Croutsch C. (director) Erstein Grasweg-PAE. Les occupations de la fin du IIIe millénaire et de l'âge du Bronze. Rapport final d'opération. Sélestat : PAIR, 2008, p. 133-135.
[8] Croutsch C. 2008 : Notice scientifique. In : Croutsch C. (director) Erstein Grasweg-PAE. Les occupations de la fin du IIIe millénaire et de l'âge du Bronze. Rapport final d'opération. Sélestat : PAIR, 2008. p. 11.
[9] Putelat O. Archéozoologie. In : Croutsch (director) Erstein Grasweg-PAE. Les occupations de la fin du IIIe millénaire et de l'âge du Bronze. Rapport final d'opération. Sélestat : PAIR, 2008, p. 61-68, 126-132, 219-226, 367-380.
[10] Putelat O. Étude archéozoologique des vestiges animaux. In : Croutsch C. (director). Les sites protohistoriques d'Erstein « Grassweg-PAE » (Alsace, Bas-Rhin) : l'occupation Rhin - Suisse – France orientale. Revue Archéologique de l'Est 60, 2011, p. 111-122.
[11] Landolt M. (director). Entzheim-Geispolsheim (Alsace, Bas-Rhin). Aéroparc (LIDL-CUS). Rapport de fouille préventive. 5 volumes. Sélestat : Pôle d'Archéologie Interdépartemental Rhénan, 2007, 182 p., 298 p., 497 p., 220 p., 353 p.
[12] Landolt M. (director). Entzheim - Geispolsheim, Bas-Rhin. Lotissement d'activités du quadrant 4. Entzheim « In der Klamm » et Geispolsheim « Schwobenfeld ». Des habitats et une nécropole néolithiques, des habitats protohistoriques, un habitat antique, un habitat et une aire funéraire mérovingiens, une position fortifiée allemande de la Première Guerre Mondiale. Rapport de fouille préventive. 6 volumes. Sélestat, Pôle d'Archéologie Interdépartemental Rhénan, 2013, 146 p., 702 p., 602 p., 200 p., 386 p., 744 p.
[13] Schneider N. 2013. Le contexte géomorphologique : des occupations au bord de la Bruche. In Landolt M. (director) Entzheim - Geispolsheim, Bas-Rhin. Lotissement d'activités du quadrant 4. Entzheim « In der Klamm » et Geispolsheim « Schwobenfeld ». Des habitats et une nécropole néolithiques, des habitats protohistoriques, un habitat antique, un habitat et une aire funéraire mérovingiens, une position fortifiée allemande de la Première Guerre Mondiale. Rapport de fouille préventive. Volume 1. Présentation générale du site. Sélestat, Pôle d'Archéologie Interdépartemental Rhénan, 2013, p. 89-139.
[14] Schaal C. L'étude carpologique. In: Landolt M. (director) Entzheim-Geispolsheim (Alsace, Bas-Rhin). Aéroparc (LIDL-CUS). Rapport de fouille préventive. Volume 1. La localisation géographique, les découvertes anciennes du secteur, les conditions d'intervention, les méthodes d'étude et les études transversales. Sélestat, Pôle d'Archéologie Interdépartemental Rhénan, 2007, p. 69-78.
[15] Schaal C. Les restes végétaux. In: Landolt M. (director). Entzheim - Geispolsheim, Bas-Rhin. Lotissement d'activités du quadrant 4. Entzheim « In der Klamm » et Geispolsheim « Schwobenfeld ». Des habitats et une nécropole néolithiques, des habitats protohistoriques, un habitat antique, un habitat et une aire funéraire mérovingiens, une position fortifiée allemande

de la Première Guerre Mondiale. Rapport de fouille préventive. Vol. 3. Les occupations protohistoriques. Sélestat, Pôle d’Archéologie Interdépartemental Rhénan, 2013, p. 445-463.
[16] Putelat O. La faune. In : Landolt M. (director) Entzheim-Geispolsheim (Alsace, Bas-Rhin). Aéroparc (LIDL-CUS). Rapport de fouille préventive. Vol. 3. Les occupations protohistoriques. Sélestat : Pôle d’Archéologie Interdépartemental Rhénan, 2007, p. 304-325.
[17] Putelat O. Geispolsheim (Bas-Rhin) « Schwobenfeld ». Archéozoologie. In : Landolt M. (director). Entzheim - Geispolsheim, Bas-Rhin. Lotissement d’activités du quadrant 4. Entzheim « In der Klamm » et Geispolsheim « Schwobenfeld ». Des habitats et une nécropole néolithiques, des habitats protohistoriques, un habitat antique, un habitat et une aire funéraire mérovingiens, une position fortifiée allemande de la Première Guerre Mondiale. Rapport de fouille préventive. Sélestat, Pôle d’Archéologie Interdépartemental Rhénan, 2013. Vol. 1, p. 71-77, Vol. 3, p. 110, 323-328, Vol. 4, p. 32, 64, 72, 82, 170.
[18] Châtelet M. Marlenheim « Hofstatt » (Bas-Rhin). Des inhumations en silo néolithiques au quartier artisanal carolingien. Rapport final d’opération. Vol.1.. Dijon, Sélestat et Strasbourg : Inrap, Pôle d’Archéologie Interdépartemental Rhénan, SRA Alsace, 2009. 271 p.
[19] Wiethold J. Agriculture, alimentation végétale et paléo-environnement à Marlenheim à la période du haut Moyen Âge. In *:* Chatelet M. (director) Dans l’environnement d’une résidence royale : Marlenheim et son territoire aux époques mérovingienne et carolingienne. Projet collectif de recherche 2012-2016. Rapport de 2e année (novembre 2013). Strasbourg : Inrap, 2013, p. 167-200.
[20] Putelat O. Les relations homme-animal dans le monde des vivants et des morts. Étude archéozoologique des établissements et des regroupements funéraires ruraux de l’Arc jurassien et de la Plaine d’Alsace, de la fin de l’Antiquité tardive au premier Moyen Âge. Thesis Université de Paris 1 Panthéon-Sorbonne, 2015, 1045 p. et 732 fig. DOI: 10.13140/RG.2.1.5115.1202.
[21] Logel T., Aoustin D., Errera M., Esteban 1., Pétrequin P., Putela O., Queyras M., Schaal C., Thomann A., Trautmann M., Vigreux T. Ostheim, Haut-Rhin, Birgelsgaerten, RD 416-rue de Strasbourg. Rapport de fouille préventive. Vol. 1. La Pré-et Protohistoire. Sélestat : Pôle d’Archéologie Interdépartemental Rhénan, 2013, 240 p.
[22] Aoustin D. Étude palynologique. In : Logel T., director. Ostheim, Haut-Rhin, Birgelsgaerten, RD 416-rue de Strasbourg. Rapport de fouille préventive. Vol. 1. La Pré-et Protohistoire. Sélestat : Pôle d’Archéologie Interdépartemental Rhénan, 2013. pp 102-109.
[23] Sell Y., Berchtold JP., Callot H., Hoff M., Gall JC., Walter JM. - L’Alsace et les Vosges. Géologie, milieux naturels, flore et faune. Lausanne/Paris : Delachaux et Niestlé, 1998, 352 p.
[24] Schaal C. Carpologie. In : Logel T. (director). Ostheim, Haut-Rhin, Birgelsgaerten, RD 416-rue de Strasbourg. Rapport de fouille préventive. Vol. 2. Le haut Moyen Âge et la période moderne. Sélestat : Pôle d’Archéologie Interdépartemental Rhénan, 2013, p. 191-208.
[25] Putelat O., Guthmann E. Première note sur les ossements animaux. In : Logel T. (director) Ostheim, Haut-Rhin, Birgelsgaerten, RD 416-rue de Strasbourg. Rapport de fouille préventive. Vol. 2. Le haut Moyen Âge et la période moderne. Sélestat : Pôle d’Archéologie Interdépartemental Rhénan, 2013, p. 161-190.
[26] Putelat O., Logel T., Bocherens H., Geigl EM ; De Lima Guimaraes S., Metz B. Une chasse aristocratique dans le Ried Centre-Alsace au premier Moyen Âge. L’apport de l’archéozoologie à la connaissance du site d’Ostheim « Birgelsgaerten » (Haut-Rhin, France). In Peytremann E. (director) Des Fleuves et des Hommes à l'époque merovingienne. Territoire fluvial et société au premier Moyen Age (V^{e}-XIIe siècle). Actes des 33$^{\textit{èmes}}$ journées internationales d’Archéologie mérovingienne. Strasbourg, 28-30 septembre 2012. Revue Archéologique de l'Est, supplément n° 42, 2016, p. 255-270.

[27] Kammerer O. Entre Vosges et Forêt-Noire : Pouvoirs, terroirs et villes de l'Oberrhein 1250-1350. Paris : Publications de la Sorbonne, 2001, 449 p.
