# Supplementary material for: Genetic diversity, genetic structure and diet of ancient and contemporary red deer (Cervus elaphus L.) from north-eastern France
Source: PLoS One. 2018 Jan 5;13(1):e0189278. doi: 10.1371/journal.pone.0189278 (PMC5755736; doi:10.1371/journal.pone.0189278)
Supplement: S1 Table — Values in italics (CER17, CER 23) correspond to collagen with chemical composition outside the reliability range, therefore the δ13C and δ15N values are not considered for palaeobiological implications. (PDF) [file pone.0189278.s002.pdf]

Lab.-N°	Archaeological Code	Location	Archaeological site	Geomorphology	Chronology	Element	cytb	d-loop	Clade	Haplotype	% N bone	%C coll	%N coll	C/N	$\delta^{13}C$	$\delta^{15}N$
CER17	108-S13	Rosheim	Lotissement Sainte Odile	Loessic terrace, eastern border of the Vosges	Neolithic (Rubané IVb, near 5100 BC)	metatarsus	removed	removed	unknown	unknown	1,60	*15,0*	*4,8*	*3,7*	*-23,2*	*4,7*
CER18	108-S19					metacarpus	Yes	Yes	A	AM235	1,14	34,5	12,2	3,3	-21,8	6,5
CER19	108-S19					ulna	Yes	Yes	A	AM235	0,76	collagen not extracted, %N too low				
CER20	108-S19					tibia	Yes	Yes	A	AM6	1,39	36,6	13,0	3,3	-23,9	5,3
CER21	33					radius	removed	removed	unknown	unknown	2,03	37,4	13,2	3,3	-22,5	5,2
CER22	33					phalanx 2	Yes	Yes	A	AM6	2,04	36,9	12,9	3,4	-22,7	5,7
CER23	122-5	Rosheim	Laser 1999		Neolithic (Grossgartach, near 4750 BC)	mandibula	partial	Yes	A	AM235	0,58	*13,6*	*4,5*	*3,6*	*-24,0*	*4,6*
CER1	3008-768	Erstein	Grasweg	Loessic terrace, Rhine valley	Bronze Age (Bronze final IIIa-RSFO, 1026-1010 BC)	metacarpus	Yes	Yes	A	AM235	3,11	42,5	15,0	3,3	-23,7	7,1
CER2	3008-786					mandibula	Yes	Yes	A	AM235	0,76	collagen not extracted, %N too low				
CER6	1079-1288	Entzheim-Geispolsheim	In der Klamm - Schwobenfeld	Alluvial terrace, Rhine valley	Iron Age (Hallstatt D3-La Tène A1, near 475 BC)	metatarsus	Yes	Yes	A	AM235	2,95	40,1	14,2	3,3	-21,7	6,3
CER7	1079-1289					maxilla	Yes	Yes	A	AM235	2,76	39,8	14,0	3,3	-21,0	5,2
CER8	1238-1266					os naviculare	Yes	Yes	A	AM235	1,23	34,1	12,0	3,3	-21,0	6,5
CER9	1238-1267					maxilla	removed	removed	unknown	unknown	2,67	40,8	14,6	3,3	-21,3	5,1
CER10	1238-1268					mandibula	Yes	Yes	A	AM235	1,21	34,1	12,1	3,3	-21,9	6,7
CER11	1610-910					mandibula	Yes	Yes	A	AM235	0,55	collagen not extracted, %N too low				
CER12	1640-878					maxilla	Yes	Yes	A	AM235	0,83	33,2	11,6	3,3	-22,6	7,4
CER13	604.2-568	Marlenheim	Hoffstatt 2007	Hill, eastern border of the Vosges	Early Middle Age (600-650 AD)	metatarsus	Yes	Yes	A	AM6	1,05	31,6	11,1	3,3	-22,4	5,4
CER14	604-391					humerus	Yes	Yes	A	AM235	0,67	collagen not extracted, %N too low				
CER15	604-392					radius	Yes	Yes	A	AM235	0,78	collagen not extracted, %N too low				
CER16	604-393					ulna	Yes	Yes	A	AM235	0,89	29,0	10,0	3,4	-22,6	6,0
CER3	3070-1167	Ostheim	Birgelsgaerten	Alluvial cone , eastern border of the Vosges	Early Middle Age (650-750 AD)	mandibula	Yes	Yes	A	AM235	1,84	40,3	14,3	3,3	-20,8	8,6
CER4	3325-479					metatarsus	Yes	Yes	A	AM6	2,41	40,4	14,3	3,3	-22,0	4,3
CER5	3325.1-2-526					calcaneus	Yes	Yes	A	AM6	0,93	30,7	10,8	3,3	-22,7	3,2
