# Supplementary material for: Genetic diversity, genetic structure and diet of ancient and contemporary red deer (Cervus elaphus L.) from north-eastern France
Source: PLoS One. 2018 Jan 5;13(1):e0189278. doi: 10.1371/journal.pone.0189278 (PMC5755736; doi:10.1371/journal.pone.0189278)
Supplement: S2 Table — (PDF) [file pone.0189278.s003.pdf]

Location and haplotypes of modern red deer analyzed in this study						Haplo-type	Haplo-type	composed haplotype	nAMe of composed haplotype
Sample	Location	Geographic unity	Commune	Department	Sex	cyt b	d-loop	cyt b/d-loop	cyt b/d-loop
DEER 096	Cornimont	"Cornim"	Cornimont	Vosges (88)	NO DATA	AVc3	AVd3	AVc3/AVd3	AM3
DEER 097	Cornimont	"Cornim"	Cornimont		NO DATA	AVc3	AVd3	AVc3/AVd3	AM3
DEER 098	Cornimont	"Cornim"	Cornimont		NO DATA	AVc3	AVd3	AVc3/AVd3	AM3
DEER 107	Cornimont	"Cornim"	Cornimont		NO DATA	AVc3	unknown	AVc3/unknown	likely AM3
DEER 109	Cornimont	"Cornim"	Cornimont		NO DATA	AVc3	unknown	AVc3/unknown	likely AM3
DEER 110	Cornimont	"Cornim"	Cornimont		NO DATA	AVc3	AVd3	AVc3/AVd3	AM3
DEER 111	Cornimont	"Cornim"	Cornimont		NO DATA	AVc2	AVd4	AVc2/AVd4	AM5
DEER 112	Cornimont	"Cornim"	Cornimont		NO DATA	AVc3	AVd3	AVc3/AVd3	AM3
DEER 113	Cornimont	"Cornim"	Cornimont		NO DATA	AVc3	AVd3	AVc3/AVd3	AM3
DEER 115	Cornimont	"Cornim"	Cornimont		NO DATA	AVc3	AVd3	AVc3/AVd3	AM3
DEER 108	Cornimont	"Cornim"[1]	Cornimont		FEMALE	AVc3	AVd3	AVc3/AVd3	AM3
DEER 094	Cornimont	"CornimNA"	Cornimont		NO DATA	AVc3	AVd3	AVc3/AVd3	AM3
DEER 095	Cornimont	"CornimNA"	Cornimont		NO DATA	AVc3	AVd3	AVc3/AVd3	AM3
DEER 100	Cornimont	"CornimNA"	Cornimont		NO DATA	AVc2	AVd2	AVc2/AVd2	AM2
DEER 101	Cornimont	"CornimNA"	Cornimont		NO DATA	AVc2	unknown	AVc2/unknown	likely AM2
DEER 102	Cornimont	"CornimNA"	Cornimont		NO DATA	AVc3	AVd3	AVc3/AVd3	AM3
DEER 103	Cornimont	"CornimNA"	Cornimont		NO DATA	AVc2	AVd2	AVc2/AVd2	AM2
DEER 104	Cornimont	"CornimNA"	Cornimont		NO DATA	AVc2	unknown	AVc2/unknown	likely AM2
DEER 105	Cornimont	"CornimNA"	Cornimont		NO DATA	AVc2	AVd2	AVc2/AVd2	AM2
DEER 106	Cornimont	"CornimNA"	Cornimont		NO DATA	AVc3	AVd3	AVc3/AVd3	AM3
DEER 114	Cornimont	"CornimNA"	Cornimont		NO DATA	AVc3	AVd3	AVc3/AVd3	AM3
DEER 099	Cornimont	"CornimNA"[2]	Cornimont		NO DATA	AVc2	AVd2	AVc2/AVd2	AM2
DEER 064	Massif 25: Femonville	"Donon Massif"	Frémonville	Meurthe-et-Moselle (54)	MALE	AVc3	AVd3	AVc3/AVd3	AM3
DEER 041	Massif 26: Domevre sur Vezouze	"Donon Massif"	Domèvre-sur-Vezouze		FEMALE	AVc3	AVd3	AVc3/AVd3	AM3
DEER 044	Massif 26: Domevre sur Vezouze	"Donon Massif"	Domèvre-sur-Vezouze		FEMALE	AVc3	AVd3	AVc3/AVd3	AM3
DEER 060	Massif 26: Herbeville	"Donon Massif"	Herbéviller		FEMALE	AVc3	AVd3	AVc3/AVd3	AM3
DEER 066	Massif 26: Neuville les badonviller	"Donon Massif"	Neuviller-lès-Badonviller		MALE	AVc3	AVd3	AVc3/AVd3	AM3
DEER 031	Massif 27: Val-et-Chatillon	"Donon Massif"	Val-et-Châtillon		FEMALE	AVc3	unknown	AVc3/unknown	likely AM3
DEER 034	Massif 27: Val- et- Chatillon	"Donon Massif"	Val-et-Châtillon		FEMALE	AVc3	AVd3	AVc3/AVd3	AM3
DEER 042	Massif 27: Cirey sur Vezouze	"Donon Massif"	Cirey-sur-Vezouze		MALE	AVc3	AVd3	AVc3/AVd3	AM3
DEER 045	Massif 27: Val- et- Chatillon	"Donon Massif"	Val-et-Châtillon		MALE	AVc3	AVd3	AVc3/AVd3	AM3
DEER 046	Massif 27: Cirey sur Vezouze	"Donon Massif"	Cirey-sur-Vezouze		FEMALE	AVc3	AVd3	AVc3/AVd3	AM3
DEER 048	Massif 27: Cirey sur Vezouze	"Donon Massif"	Cirey-sur-Vezouze		FEMALE	AVc3	AVd3	AVc3/AVd3	AM3
DEER 049	Massif 27: Cirey sur Vezouze	"Donon Massif"	Cirey-sur-Vezouze		MALE	AVc3	AVd3	AVc3/AVd3	AM3
DEER 051	Massif 27: Val-et-Chatillon	"Donon Massif"	Val-et-Châtillon		FEMALE	AVc3	AVd3	AVc3/AVd3	AM3
DEER 052	Massif 27: Val-et-Chatillon	"Donon Massif"	Val-et-Châtillon		MALE	unknown3	AVd3	unknown/AVd3	likely AM3

Sample	Location	Geographic unity	Commune	Department	Sex	cyt *b*	d-loop	cyt *b* /d-loop	cyt *b* /d-loop
DEER 054	Massif 27: Val-et-Chatillon	"Donon Massif"	Val-et-Châtillon	Meurthe-et-Moselle (54)	FEMALE	AVc3	AVd3	AVc3/AVd3	AM3
DEER 056	Massif 27: Cirey sur Vezouze	"Donon Massif"	Cirey-sur-Vezouze		MALE	AVc3	AVd3	AVc3/AVd3	AM3
DEER 057	Massif 27: Val-et-Chatillon	"Donon Massif"	Val-et-Châtillon		FEMALE	AVc3	AVd3	AVc3/AVd3	AM3
DEER 061	Massif 27: Val-et-Chatillon	"Donon Massif"	Val-et-Châtillon		MALE	AVc3	AVd3	AVc3/AVd3	AM3
DEER 062	Massif 27: Val-et-Chatillon	"Donon Massif"	Val-et-Châtillon		MALE	AVc3	AVd3	AVc3/AVd3	AM3
DEER 063	Massif 27: Cirey sur Vezouze	"Donon Massif"	Cirey-sur-Vezouze		NO DATA	unknown	AVd3	unknown/AVd3	likely AM3
DEER 065	Massif 27: Val-et-Chatillon	"Donon Massif"	Val-et-Châtillon		MALE	AVc3	AVd3	AVc3/AVd3	AM3
DEER 069	Massif 27: Val-et-Chatillon	"Donon Massif"	Val-et-Châtillon		MALE	AVc3	AVd3	AVc3/AVd3	AM3
DEER 035	Massif 28: Sainte Pole	"Donon Massif"	Sainte-Pôle		MALE	AVc3	AVd3	AVc3/AVd3	AM3
DEER 038	Massif 30: Brouville	"Donon Massif"	Brouville		MALE	AVc3	AVd3	AVc3/AVd3	AM3
DEER 039	Massif 30: Orgeval	"Donon Massif"	Ogéviller		MALE	AVc3	AVd3	AVc3/AVd3	AM3
DEER 068	Massif 30: Hablainville	Between Donon and Parroy	Hablainville		NO DATA	AVc2	AVd2	AVc2/AVd2	AM2
DEER 116	Bresse	"La Bresse"	La Bresse	Vosges (88)	NO DATA	AVc3	AVd3	AVc3/AVd3	AM3
DEER 117	Bresse	"La Bresse"	La Bresse		NO DATA	AVc3	AVd3	AVc3/AVd3	AM3
DEER 070	La Petite Pierre (Réserve de chasse)	"La Petite Pierre"	La Petite Pierre	Bas-Rhin (67)	NO DATA	AVc3	AVd3	AVc3/AVd3	AM3
DEER 071	La Petite Pierre (Réserve de chasse)	"La Petite Pierre"	La Petite Pierre		FEMALE	AVc3	unknown	AVc3/unknown	likely AM3
DEER 072	La Petite Pierre (Réserve de chasse)	"La Petite Pierre"	La Petite Pierre		NO DATA	AVc3	AVd3	AVc3/AVd3	AM3
DEER 073	La Petite Pierre (Réserve de chasse)	"La Petite Pierre"	La Petite Pierre		NO DATA	AVc3	AVd3	AVc3/AVd3	AM3
DEER 074	La Petite Pierre (Réserve de chasse)	"La Petite Pierre"	La Petite Pierre		NO DATA	AVc3	AVd3	AVc3/AVd3	AM3
DEER 075	La Petite Pierre (Réserve de chasse)	"La Petite Pierre"	La Petite Pierre		NO DATA	AVc3	unknown	AVc3/unknown	likely AM3
DEER 076	La Petite Pierre (Réserve de chasse)	"La Petite Pierre"	La Petite Pierre		FEMALE	unknown	unknown	unknown	unknown
DEER 077	La Petite Pierre (Réserve de chasse)	"La Petite Pierre"	La Petite Pierre		NO DATA	AVc3	AVd3	AVc3/AVd3	AM3
DEER 078	La Petite Pierre (Réserve de chasse)	"La Petite Pierre"	La Petite Pierre		NO DATA	AVc3	AVd3	AVc3/AVd3	AM3
DEER 079	La Petite Pierre (Réserve de chasse)	"La Petite Pierre"	La Petite Pierre		NO DATA	AVc3	AVd3	AVc3/AVd3	AM3
DEER 080	La Petite Pierre (Réserve de chasse)	"La Petite Pierre"	La Petite Pierre		FEMALE	unknown	unknown	unknown	unknown
DEER 081	La Petite Pierre (Réserve de chasse)	"La Petite Pierre"	La Petite Pierre		NO DATA	AVc3	AVd3	AVc3/AVd3	AM3
DEER 082	La Petite Pierre (Réserve de chasse)	"La Petite Pierre"	La Petite Pierre		NO DATA	AVc3	AVd3	AVc3/AVd3	AM3
DEER 083	La Petite Pierre (Réserve de chasse)	"La Petite Pierre"	La Petite Pierre		NO DATA	unknown	AVd3	unknown/AVd3	likely AM3
DEER 084	La Petite Pierre (Réserve de chasse)	"La Petite Pierre"	La Petite Pierre		NO DATA	AVc3	AVd3	AVc3/AVd3	AM3
DEER 085	La Petite Pierre (Réserve de chasse)	"La Petite Pierre"	La Petite Pierre		NO DATA	AVc3	unknown	AVc3/unknown	likely AM3
DEER 086	La Petite Pierre (Réserve de chasse)	"La Petite Pierre"	La Petite Pierre		NO DATA	AVc3	unknown	AVc3/?	likely AM3
DEER 087	La Petite Pierre (Réserve de chasse)	"La Petite Pierre"	La Petite Pierre		FEMALE	AVc3	AVd3	AVc3/AVd3	AM3
DEER 088	La Petite Pierre (Réserve de chasse)	"La Petite Pierre"	La Petite Pierre		FEMALE	AVc3	AVd3	AVc3/AVd3	AM3
DEER 089	La Petite Pierre (Réserve de chasse)	"La Petite Pierre"	La Petite Pierre		FEMALE	AVc3	AVd3	AVc3/AVd3	AM3
DEER 090	La Petite Pierre (Réserve de chasse)	"La Petite Pierre"	La Petite Pierre		NO DATA	AVc3	unknown	AVc3/unknown	likely AM3
DEER 091	La Petite Pierre (Réserve de chasse)	"La Petite Pierre"	La Petite Pierre		FEMALE	AVc3	AVd3	AVc3/AVd3	AM3
DEER 092	La Petite Pierre (Réserve de chasse)	"La Petite Pierre"	La Petite Pierre		NO DATA	AVc3	AVd3	AVc3/AVd3	AM3
DEER 093	La Petite Pierre (Réserve de chasse)	"La Petite Pierre"	La Petite Pierre		NO DATA	unknown	unknown	unknown	unknown

Sample	Location	Geographic unity	Commune		Sex	cyt *b*	d-loop	cyt *b* /d-loop	cyt *b* /d-loop
DEER 032	Massif 24: Embermenil	"Parroy Massif"	Emberménil	Meurthe-et-Moselle (54)	FEMALE	AVc2	AVd2	AVc2/AVd2	AM2
DEER 033	Massif 24: Laneuvevile aux Bois	"Parroy Massif"	Laneuveville-aux-Bois		FEMALE	AVc2	AVd2	AVc2/AVd2	AM2
DEER 036	Massif 24: Embermenil	Between Donon and Parroy	Emberménil		MALE	AVc3	AVd3	AVc3/AVd3	AM3
DEER 037	Massif 24: Croismare	"Parroy Massif"	Croismare		FEMALE	AVc2	AVd2	AVc2/AVd2	AM2
DEER 040	Massif 24: Embermenil	"Parroy Massif"	Emberménil		MALE	AVc2	unknown	AVc2/unknown	likely AM2
DEER 047	Massif 24: Laneuvevile aux Bois	"Parroy Massif"	Laneuveville-aux-Bois		MALE	AVc2	AVd2	AVc2/AVd2	AM2
DEER 050	Massif 24: Embermenil	"Parroy Massif"	Emberménil		FEMALE	AVc2	AVd2	AVc2/AVd2	AM2
DEER 058	Massif 24: Laneuvevile aux Bois	"Parroy Massif"	Laneuveville-aux-Bois		MALE	AVc2	AVd2	AVc2/AVd2	AM2
DEER 067	Massif 24: Laneuvevile aux Bois	Between Donon and Parroy	Laneuveville-aux-Bois		MALE	AVc3	AVd3	AVc3/AVd3	AM3
DEER 043	Massif 25: Leintrey	"Parroy Massif"	Leintrey		MALE	AVc2	AVd2	AVc2/AVd2	AM2
DEER 053	Massif 25: Remoncourt	Between Donon and Parroy	Remoncourt		MALE	AVc3	AVd3	AVc3/AVd3	AM3
DEER 055	Massif 25: Domjevin	"Parroy Massif"	Domjevin		MALE	unknown	AVd2	unknown/AVd2	likely AM2
DEER 059	Massif 25: Leintrey	"Parroy Massif"	Leintrey		FEMALE	AVc2	AVd2	AVc2/AVd2	AM2
DEER 001	Massif de Rambervillers	"Rambervillers"	Rambervillers	Vosges (88)	NO DATA	AVc1	AVd1	AVc1/AVd1	AM1
DEER 002	Massif de Rambervillers	"Rambervillers"	Rambervillers		NO DATA	unknown	unknown	unknown	unknown
DEER 003	Massif de Rambervillers	"Rambervillers"	Rambervillers		NO DATA	AVc1	AVd1	AVc1/AVd1	AM1
DEER 004	Massif de Rambervillers	"Rambervillers"	Rambervillers		NO DATA	AVc1	AVd1	AVc1/AVd1	AM1
DEER 005	Massif de Rambervillers	"Rambervillers"	Rambervillers		NO DATA	AVc1	unknown	AVc1/unknown	likely AM1
DEER 006	Massif de Rambervillers	"Rambervillers"	Rambervillers		NO DATA	AVc2	AVd2	AVc2/AVd2	AM2
DEER 007	Massif de Rambervillers	"Rambervillers"	Rambervillers		NO DATA	unknown	unknown	unknown	unknown
DEER 008	Massif de Rambervillers	"Rambervillers"	Rambervillers		NO DATA	AVc1	AVd1	AVc1/AVd1	AM1
DEER 009	Massif de Rambervillers	"Rambervillers"	Rambervillers		NO DATA	AVc1	AVd1	AVc1/AVd1	AM1
DEER 010	Massif de Rambervillers	"Rambervillers"	Rambervillers		NO DATA	AVc1	AVd1	AVc1/AVd1	AM1
DEER 011	Massif de Rambervillers	"Rambervillers"	Rambervillers		NO DATA	AVc1	AVd1	AVc1/AVd1	AM1
DEER 012	Massif de Rambervillers	"Rambervillers"	Rambervillers		NO DATA	AVc1	AVd1	AVc1/AVd1	AM1
DEER 013	Massif de Rambervillers	"Rambervillers"	Rambervillers		NO DATA	AVc1	AVd1	AVc1/AVd1	AM1
DEER 014	Massif de Rambervillers	"Rambervillers"	Rambervillers		NO DATA	unknown	AVd1	unknown/AVd1	likely AM1
DEER 015	Massif de Rambervillers	"Rambervillers"	Rambervillers		NO DATA	AVc2	AVd2	AVc2/AVd2	AM2
DEER 016	Massif de Rambervillers	"Rambervillers"	Rambervillers		NO DATA	AVc1	AVd1	AVc1/AVd1	AM1
DEER 017	Massif de Rambervillers	"Rambervillers"	Rambervillers		NO DATA	AVc1	AVd1	AVc1/AVd1	AM1
DEER 018	Massif de Rambervillers	"Rambervillers"	Rambervillers		NO DATA	unknown	AVd3	unknown/AVd3	likely AM3
DEER 019	Massif de Rambervillers	"Rambervillers"	Rambervillers		NO DATA	AVc1	AVd1	AVc1/AVd1	AM1
DEER 020	Massif de Rambervillers	"Rambervillers"	Rambervillers		NO DATA	unknown	unknown	unknown	unknown
DEER 021	Massif de Rambervillers	"Rambervillers"	Rambervillers		NO DATA	AVc1	AVd1	AVc1/AVd1	AM1
DEER 022	Massif de Rambervillers	"Rambervillers"	Rambervillers		NO DATA	AVc3	AVd3	AVc3/AVd3	AM3
DEER 023	Massif de Rambervillers	"Rambervillers"	Rambervillers		NO DATA	AVc1	AVd1	AVc1/AVd1	AM1
DEER 024	Massif de Rambervillers	"Rambervillers"	Rambervillers		NO DATA	AVc2	AVd2	AVc2/AVd2	AM2
DEER 025	Massif de Rambervillers	"Rambervillers"	Rambervillers		NO DATA	AVc2	AVd1	AVc2/AVd1	AM4
DEER 026	Massif de Rambervillers	"Rambervillers"	Rambervillers		NO DATA	AVc1	AVd1	AVc1/AVd1	AM1
DEER 027	Massif de Rambervillers	"Rambervillers"	Rambervillers		NO DATA	AVc2	AVd2	AVc2/AVd2	AM2
DEER 028	Massif de Rambervillers	"Rambervillers"	Rambervillers		NO DATA	AVc1	AVd1	AVc1/AVd1	AM1
DEER 029	Massif de Rambervillers	"Rambervillers"	Rambervillers		NO DATA	AVc1	AVd1	AVc1/AVd1	AM1
DEER 030	Massif de Rambervillers	"Rambervillers"	Rambervillers		NO DATA	AVc2	AVd2	AVc2/AVd2	AM2

**comments**
genetic sexing: male

**comments**
genetic sexing: male

**comments**
genetic sexing: female
genetic sexing: female
genetic sexing: female
