# Supplementary material for: Genetic diversity, genetic structure and diet of ancient and contemporary red deer (Cervus elaphus L.) from north-eastern France
Source: PLoS One. 2018 Jan 5;13(1):e0189278. doi: 10.1371/journal.pone.0189278 (PMC5755736; doi:10.1371/journal.pone.0189278)
Supplement: S3 Table — 2013 used for diversity calculation (Arlequin) and network construction (Network 5.001). (PDF) [file pone.0189278.s004.pdf]

Ancient Samples	MJN	Arlequin		
**Species**	**Isolate**	**Isolate**	**Genbank accession number for Cytb**	**Genbank accession number for Control Region**
C. elaphus	MM007	MM007	KF133831	KF133903
C. elaphus	MM020	MM020	KF133832	KF133904
C. elaphus	MM079	MM079	KF133833	KF133905
C. elaphus	MM080	MM080	KF133834	KF133906
C. elaphus	MM082	MM082	KF133835	KF133907
C. elaphus	MM093	MM093	KF133836	KF133908
C. elaphus	MM095	MM095	KF133837	KF133909
C. elaphus	MM098	MM098	KF133838	KF133910
C. elaphus	MM131	MM131	KF133839	KF133911
C. elaphus	MM135	MM135	KF133840	KF133912
C. elaphus	MM139	MM139	KF133841	KF133913
C. elaphus	MM150	MM150	KF133842	KF133914
C. elaphus	MM153	MM153	KF133843	KF133915
C. elaphus	MM154	MM154	KF133844	KF133916
C. elaphus	MM165	MM165	KF133845	KF133917
C. elaphus	MM175	MM175	KF133846	KF133918
C. elaphus	MM183	MM183	KF133847	KF133919
C. elaphus	MM186	MM186	KF133848	KF133920
C. elaphus	MM187	MM187	KF133849	KF133921
C. elaphus	MM190	MM190	KF133850	KF133922
C. elaphus	MM192	MM192	KF133851	KF133923
C. elaphus	MM198	MM198	KF133852	KF133924
C. elaphus	MM210	MM210	KF133853	KF133925
C. elaphus	MM214	MM214	KF133854	KF133926
C. elaphus	MM224	MM224	KF133855	KF133927
C. elaphus	MM235	MM235	KF133856	KF133928
C. elaphus	MM239	MM239	KF133857	KF133929
C. elaphus	MM245	MM245	KF133858	KF133930
C. elaphus	MM251	MM251	KF133859	KF133931
C. elaphus	MM272	MM272	KF133860	KF133932
C. elaphus	MM352	MM352	KF133861	KF133933
C. elaphus	MM379	MM379	KF133862	KF133934
C. elaphus	MM408	MM408	KF133863	KF133935
C. elaphus	MM455	MM455	KF133865	KF133937
C. elaphus	MM461	MM461	KF133866	KF133938
C. elaphus	MM464	MM464	KF133867	KF133939
C. elaphus	MM468	MM468	KF133871	KF133943
C. elaphus	MM472	MM472	KF133868	KF133940
C. elaphus	MM473	MM473	KF133869	KF133941
C. elaphus	MM475	MM475	KF133870	KF133942
C. elaphus	MM477	MM477	KF133872	KF133944
C. elaphus	MM493	MM493	KF133873	KF133945

Modern Samples	MJN	no Arlequin		
**Subspecies name**	**Isolate**	**Isolate**	**Genbank accession number for Cytb**	**Genbank accession number for Control Region**
C. e. hippelaphus	MM335	MM335	KF133877	KF133949
C. e. hippelaphus	MM702	MM702	KF133875	KF133947
C. e. maral	MM331	MM331	KF133895	KF133967
C. e. hippelaphus	MM719	MM719	KF133879	KF133951
C. e. hippelaphus	MM781	MM781	KF133890	KF133962
C. e. hippelaphus	MM754	MM754	KF133880	KF133952
C. e. scoticus	D52	D52	KF133898	KF133970
C. e. atlanticus	MM762	MM762	KF133874	KF133946
C. e. atlanticus	MM765	MM765	KF133885	KF133957
C. e. hippelaphus	MM337	MM337	KF133878	KF133950
C. e. maral	MM315	MM315	KF133892	KF133964
C. e. maral	MM313	MM313	KF133891	KF133963
C. e. scoticus	MM733	MM733	KF133897	KF133969
C. e. scoticus	MM732	MM732	KF133896	KF133968
C. e. hispanicus	MM718	MM718	KF133888	KF133960
C. e. hispanicus	MM721	MM721	KF133886	KF133958
C. e. elaphus	MM770	MM770	KF133882	KF133954
C. e. maral	MM766	MM766	KF133894	KF133966
C. e. bactrianus	MM300	MM300	KF133900	KF133972
C. e. nelsoni	MM722	MM722	KF133901	KF133973
C. nippon	MM714	MM714	KF133902	KF133974
